# Effects of *Qingjin Huatan* decoction on pulmonary function and inflammatory mediators in acute exacerbations of chronic obstructive pulmonary disease: a systematic review and meta-analysis

**DOI:** 10.3389/fphar.2024.1466677

**Published:** 2024-10-18

**Authors:** Xuqin Du, Yong Chen, Ruodai Zhang, Lipeng Shi, Yi Ren

**Affiliations:** ^1^ School of Traditional Chinese Medicine, Chongqing University of Chinese Medicine, Chongqing, China; ^2^ Department of Classic Traditional Chinese Medicine, Chongqing Traditional Chinese Medicine Hospital, Chongqing, China; ^3^ College of Traditional Chinese Medicine, Chongqing Medical University, Chongqing, China

**Keywords:** *Qingjin Huatan* decoction, acute exacerbations of chronic obstructive pulmonary disease, pulmonary function, inflammation mediators, randomized controlled trials, systematic review, meta-analysis

## Abstract

**Background:**

The inflammatory response is the main pathophysiological basis of acute exacerbations of chronic obstructive pulmonary disease (AECOPD) and is a key factor leading to frequent exacerbations and disease progression. Suppressing the inflammatory response can improve pulmonary function, prognosis, and quality of life in AECOPD patients.

**Purpose:**

To evaluate the effect of *Qingjin Huatan* decoction (QHD) on pulmonary function and inflammatory mediators in AECOPD patients.

**Methods:**

Randomized controlled trials (RCTs) on the treatment of AECOPD with QHD were retrieved from eight Chinese and English electronic databases up to 31 May 2024. The quality of the studies was assessed using the Cochrane Risk of Bias Tool and the modified Jadad scale. Statistical analysis, sensitivity analysis, and publication bias assessment were performed using Stata 17.0 software.

**Results:**

A total of 40 RCTs involving 3,475 AECOPD patients were included. Compared to conventional treatment, QHD significantly improved pulmonary function, with increases in FEV1 (MD = 0.30, 95% CI: 0.26 to 0.34, *p* = 0.000), FVC (MD = 0.34, 95% CI: 0.27 to 0.41, *p* = 0.000), FEV1/FVC (MD = 6.07, 95% CI: 5.55 to 6.58, *p* = 0.000), and PaO_2_ (MD = 7.20, 95% CI: 4.94 to 9.47, *p* = 0.000), and a decrease in PaCO_2_ (MD = −5.37, 95% CI: 7.99 to −2.74, *p* = 0.000). QHD also significantly suppressed the expression of inflammatory mediators, including TNF-α (MD = −10.87, 95% CI: 12.51 to −9.23, *p* = 0.000), IL-1β (MD = −13.63, 95% CI: −16.31 to −10.95, *p* = 0.000), IL-6 (MD = −7.58, 95% CI: −10.10 to −5.06, *p* = 0.000), IL-8 (MD = −9.45, 95% CI: −12.05 to −6.85, *p* = 0.000), CRP (MD = −5.62, 95% CI: −6.60 to −4.65, *p* = 0.000), and PCT (MD = −0.84, 95% CI: −1.07 to −0.62, *p* = 0.000). Additionally, QHD improved clinical efficacy (RR = 4.16, 95% CI: 3.26 to 5.30, *p* = 0.000) without increasing the incidence of adverse reactions (RR = 1.04, 95% CI: 0.68 to 1.61, *p* = 0.000).

**Conclusion:**

Existing evidence suggests that QHD can significantly improve pulmonary function, suppress the expression of inflammatory mediators, and enhance clinical efficacy in AECOPD patients, with a good safety profile. Given the limitations of this study, more high-quality studies are needed to provide reliable evidence.

**Systematic Review Registration:**

https://www.crd.york.ac.uk/PROSPERO/display_record.php?RecordID=559436, identifier CRD42024559436

## 1 Introduction

Chronic obstructive pulmonary disease (COPD) is a common, preventable, and treatable heterogeneous lung disease characterized by progressively worsening airflow limitation and chronic respiratory symptoms such as cough, sputum production, and dyspnea (Celli et al., 2022). Between 2009 and 2019, the global mortality rate of COPD rose by 35.4%, and it is expected to become the third leading cause of death by 2030 (Al Wachami et al., 2024). Acute exacerbations of chronic obstructive pulmonary disease (AECOPD) significantly contribute to the overall mortality of COPD and have a detrimental impact on patients’ quality of life, disease progression, and socioeconomic burden (Boers et al., 2023). Current preventive and control strategies for AECOPD include smoking cessation, exercise, vaccination, pulmonary rehabilitation, and pharmacotherapy (Wedzicha et al., 2017). Despite these measures, many stable COPD patients continue to experience frequent exacerbations, which severely affect their quality of life and increase the economic burden (Tashkin et al., 2024). Therefore, reducing the frequency of acute exacerbations is crucial for improving symptoms, pulmonary function, and prognosis in AECOPD patients.

AECOPD is characterized by an inflammatory response, worsened by bacteria, viruses, and air pollution (D’Anna et al., 2021). Multiple inflammatory cells, cytokines, and mediators play crucial roles in AECOPD. Inflammation is central to AECOPD development and is closely linked to declining pulmonary function and increased mortality (Hatipoglu and Aboussouan, 2016; Stevermer et al., 2021). Persistent inflammation in the airways, lung parenchyma, and pulmonary microvasculature is a key pathogenic mechanism (Hatipoglu and Aboussouan, 2016). Macrophages and neutrophils are the primary inflammatory cells involved in AECOPD (Xu et al., 2023). These cells, when stimulated by harmful substances like cigarette smoke and dust, release inflammatory mediators such as TNF-α, IL-1β, and IL-6. These mediators act on airway epithelial cells, inducing goblet cell metaplasia and excessive mucus secretion (Chung et al., 2024). Furthermore, inflammatory responses stimulate epithelial cells to release growth factors, promoting the proliferation of airway smooth muscle and fibroblasts, which leads to small airway remodeling (Wang et al., 2018). Matrix metalloproteinases from macrophages and elastases from neutrophils destroy elastin in pulmonary connective tissue, damaging the alveolar epithelium and causing irreversible pulmonary injury and emphysema (Christopoulou et al., 2023). Targeting or regulating inflammation can effectively reduce AECOPD frequency and mortality (Yousuf et al., 2021). Therefore, inhibiting the inflammatory response is crucial for alleviating symptoms, improving pulmonary function, and enhancing prognosis in AECOPD patients.

*Qingjin Huatan* decoction (QHD) is a classic traditional Chinese medicine (TCM) formula, originating from Ming Dynasty physician Ye Wenling’s “*Genera Principles of Medicine*”. Comprising *Scutellaria baicalensis* Georgi (Huangqin), *Gardenia jasminoides* Ellis (Zhizi), *Platycodon grandiflorum* (Jacq.) A. DC. (Jiegeng), *Ophio pogon japonicus* (L.f). Ker-Gawl. (Maidong), *Morus alba* L. (Sangbaipi), *Fritillaria thunbergii* Miq. (Zhebeimu), *Anemarrhena asphodeloides* Bge. (Zhimu), *Trichosanthes kirilowii* Maxim. (Gualoupi), *Citrus reticulata* Blanco (Juhong), *Poria cocos* (Schw.) Wolf (Fuling), and *Glycyrrhiza uralensis* Fisch. (Gancao), QHD is known for its abilities to clear lung heat, resolve phlegm, and relieve cough and asthma. Historically, it has been widely used in treating excessive phlegm, cough, and wheezing caused by lung heat. In the treatment of AECOPD, QHD has become a popular formula among TCM practitioners due to its significant anti-inflammatory, expectorant, antitussive, bronchodilatory, and immunomodulatory effects (Yang et al., 2023a). Modern pharmacological studies further validate the diverse therapeutic properties of QHD. It has been shown to regulate key signaling pathways such as JAK/STAT (Zhao et al., 2019; Liu M. et al., 2023), ERK/p38 MAPK (Chen et al., 2016), and p38 MAPK/NF-κB (Meng et al., 2019), contributing to its anti-inflammatory effects. Additionally, QHD has been found to modulate autophagy-related mechanisms, offering protective effects in viral pneumonia models (Pan et al., 2020; Wu et al., 2019). These findings highlight the broad clinical potential of QHD in treating respiratory diseases. QHD is widely used in China to treat AECOPD, community-acquired pneumonia, acute and chronic bronchitis, and bronchiectasis, showing considerable efficacy (Zhang Q. L. et al., 2021). However, there is a lack of comprehensive evaluations on its effects on pulmonary function and inflammatory mediators in AECOPD patients. This study aims to fill this gap by conducting a systematic review and meta-analysis of randomized controlled trials (RCTs) to assess the clinical efficacy of QHD in AECOPD management.

## 2 Methods

### 2.1 Systematic review registration

This meta-analysis adhered to the PRISMA (Preferred Reporting Items for Systematic Reviews and Meta-Analyses) guidelines (Hutton et al., 2015) (Supplementary Material S1) and was registered in the PROSPERO database under Registration Number: CRD42024559436.

### 2.2 Composition and taxonomic authentication of QHD

The botanical ingredients of QHD have been validated through authoritative taxonomic resources such as the Medicinal Plant Names Services (MPNS) and Plants of the World Online (POWO). Complete botanical names, including their authorities, family classifications, and relevant pharmacopeial references, are provided in Supplementary Material S2.

### 2.3 Database and search strategy

Two reviewers (XD and LS) independently conducted the literature search. As of 31 May 2024, we performed a comprehensive search of eight electronic databases: Web of Science, Embase, PubMed, Cochrane Library, China National Knowledge Infrastructure (CNKI), Wanfang Data, Chinese Science and Technology Journals Database (CQVIP), and China Biology Medicine Database (CBM). Additionally, we searched the Chinese Clinical Trial Registry to identify potential studies.

The search strategy combined MeSH terms and free-text terms. Search terms included, but were not limited to, “acute exacerbations of chronic obstructive pulmonary disease or AECOPD”, “chronic obstructive pulmonary disease or COPD”, “pulmonary function”, “inflammatory mediators”, “*Qingjin Huatan* decoction”, and “*Qingjin Huatan* tang”. There were no restrictions on region, language, or publication type. The detailed search strategy is provided in Supplementary Material S3.

### 2.4 Inclusion and exclusion criteria

(1) Inclusion criteria
 (I) Study design: Randomized controlled trials (RCTs).
 (II) Participants: Patients diagnosed with AECOPD based on pulmonary function tests according to the GOLD guidelines.
 (III) Intervention: Combination therapy of QHD and conventional treatment (CT).
 (IV) Control: Placebo or conventional treatment.
 (V) Outcome measures:

Primary outcomes: Pulmonary function indicators (FEV1, FVC, FEV1/FVC, PaO2, PaCO2) and inflammatory mediators (TNF-α, IL-1β, IL-6, IL-8, CRP, PCT).

Secondary outcomes: Clinical efficacy and adverse reactions.

(2) Exclusion criteria
 (I) Non-randomized controlled trials.
 (II) Patients with stable COPD.
 (III) Treatment groups containing other traditional Chinese medicine preparations besides QHD.
 (IV) Duplicate publications, with only studies containing complete data being included.
 (V) Studies without primary outcome measures or those for which data could not be obtained.

### 2.5 Data extraction

After removing duplicates using Endnote 20 software, two reviewers (XD and LS) independently assessed the studies for inclusion and extracted data. The data were stored in a pre-designed Excel spreadsheet, which included the following information: first author, publication year, article title, sample size, gender, age, duration of COPD, intervention drugs and treatment duration, and outcome measures. Any discrepancies or differences during the assessment were resolved through discussion with a third reviewer (YR) to reach a consensus.

### 2.6 Quality assessment

Two reviewers (YC and RZ) independently evaluated the quality of the included studies using the Cochrane Risk of Bias Tool (Cumpston et al., 2019) (Cochrane Handbook V.5.1.0) and the modified Jadad scale (Lunny et al., 2022). The Cochrane Risk of Bias Tool assesses seven domains: random sequence generation, allocation concealment, blinding of participants and personnel, blinding of outcome assessment, incomplete outcome data, selective reporting, and other biases. Each domain is rated as “high risk”, “low risk”, or “unclear risk”. The modified Jadad scale includes four domains: random sequence generation, allocation concealment, blinding, and withdrawals/dropouts, with respective scores of 2, 2, 2, and 1. Trials scoring 1 to 3 are considered low quality, while those scoring 4 to 7 are considered high quality. Any disagreements were resolved by involving a third reviewer (YR) for discussion.

### 2.7 Data analysis

Statistical analysis was performed using Stata 17.0. For dichotomous data, the risk ratio (RR) was calculated, while for continuous data, the mean difference (MD) was computed. The *I* ^2^ statistic was used to assess heterogeneity among the studies. If heterogeneity was low (*p* > 0.05, *I* ^*2*^ < 50%), a fixed-effects model was applied; otherwise, a random-effects model was used. Subgroup analysis was conducted based on the duration of the treatment. Sensitivity analysis was performed by sequentially excluding each study. Additionally, publication bias was evaluated using Begg’s and Egger’s tests, and a funnel plot was used for visualization.

## 3 Results

### 3.1 Search results and study characteristics

**FIGURE 1:**
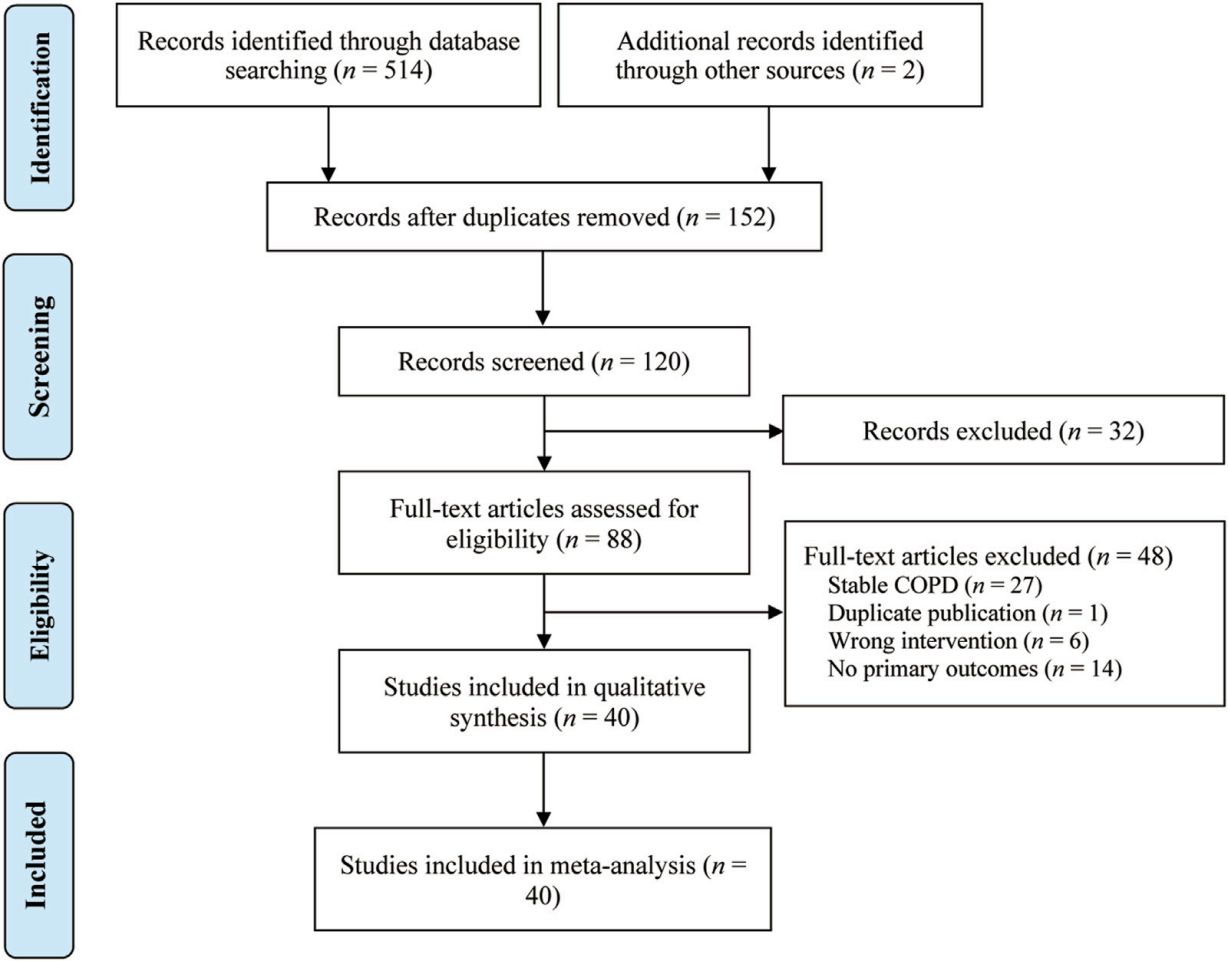
The PRISMA study flowchart.

The study characteristics of the 40 included RCTs (Chen et al., 2014; Chen and Huang, 2015; Geng et al., 2023; Guo, 2018; Hu and Zhao, 2018; Huang et al., 2022; Huo et al., 2022; Jiang and Liu, 2019; Jiang and Chen, 2017; Li et al., 2014; Li et al., 2021; Liu et al., 2021; Liu L. et al., 2023; Ni, 2021; Qin, 2022; Sun, 2020; Tang et al., 2020; Wang, 2019; Wang, 2022; Wei, 2020; Wei and Niu, 2017; Wen, 2022; Wu, 2014; Xie, 2016; Yang et al., 2023b; Yu et al., 2022; Yu, 2019; Yuan, 2018; Zhang B. R., 2018; Zhang F., 2018; Zhang G. et al., 2021; Zhang and Li, 2020; Zhang and Ge, 2022; Zhang et al., 2016; Zhang et al., 2015; Zhao, 2016; Zhao, 2023; Zhou et al., 2014; Zhou et al., 2016; Zhu, 2021) are summarized in Table 1. These studies, published between 2014 and 2023, were conducted in China and encompassed a total of 3,475 patients (2,009 males and 1,466 females) with AECOPD, with sample sizes ranging from 29 to 93. The control groups received standard treatment for AECOPD as recommended by the GOLD guidelines, which includes oxygen therapy, anti-infection measures, cough relief, and bronchodilation. The treatment groups received a combination of QHD and standard treatment. The reported outcomes across the included studies were: FEV1 (28 studies), FVC (30 studies), FEV1/FVC (29 studies), PaO_2_ (10 studies), PaCO_2_ (9 studies), TNF-α (15 studies), IL-1β (8 studies), IL-6 (7 studies), IL-8 (10 studies), CRP (13 studies), PCT (8 studies), clinical efficacy (35 studies) and adverse reactions (10 studies). The selection process of the studies is illustrated in Figure 1.

**TABLE 1:** Included studies basic characteristics.

Study ID	Sample size		Sex (M/F)		Mean age (years)		COPD course (years)		Interventions		Treatment duration	Outcomes
	T	C	T	C	T	C	T	C	T	C		
Chen et al. (2014)	60	60	24/36	32/28	66.5 ± 5.7	66.3 ± 5.8	6.4 ± 1.8	6.3 ± 1.7	QHD + CT	CT	10 d	①②③⑫
Chen and Huang (2015)	29	29	13/16	15/14	64.94 ± 12.37	63.42 ± 11.26	1.21 ± 0.97	1.24 ± 0.89	QHD + CT	CT	21 d	③⑫
Geng et al. (2023)	50	50	28/22	26/24	68.47 ± 5.04	67.58 ± 4.97	5.18 ± 1.68	5.23 ± 1.71	QHD + CT	CT	10 d	①②③⑫
Guo (2018)	42	42	25/17	24/18	60.94 ± 7.05	61.37 ± 7.28	7.58 ± 1.62	7.36 ± 1.57	QHD + CT	CT	10 d	①②③⑫
Hu and Zhao (2018)	36	36	22/14	26/10	65.18 ± 3.41	64.23 ± 3.17	—	—	QHD + CT	CT	28 d	①②③⑥⑨⑫⑬
Huang et al. (2022)	43	43	22/21	20/23	65.89 ± 3.92	66.01 ± 3.02	—	—	QHD + CT	CT	7 d	①②③⑥⑨⑫
Huo et al. (2022)	40	40	24/16	23/17	71.15 ± 2.13	71.32 ± 2.14	7.55 ± 1.24	7.69 ± 1.23	QHD + CT	CT	14 d	⑧⑩⑪⑬
Jiang and Liu (2019)	41	40	23/18	24/16	72.43 ± 8.22	70.65 ± 8.98	4.05 ± 1.53	4.75 ± 1.89	QHD + CT	CT	7 d	⑥⑦⑫
Jiang and Chen (2017)	43	43	26/17	24/19	56.43 ± 6.53	56.43 ± 6.53	8.43 ± 4.36	8.01 ± 4.42	QHD + CT	CT	10 d	①②③④⑤⑫
Li et al. (2014)	40	40	28/12	23/17	55.05 ± 5.03	55.10 ± 4.90	9.05 ± 1.05	9.10 ± 1.10	QHD + CT	CT	14 d	①②③⑧⑩⑫
Li et al. (2021)	58	40	34/24	23/17	67.74 ± 5.56	64.42 ± 6.02	7.45 ± 2.35	7.56 ± 2.78	QHD + CT	CT	7 d	①②③④⑤⑦⑧⑩⑫⑬
Liu et al. (2021)	30	30	21/9	19/11	72.43 ± 8.22	70.65 ± 8.9	4.05 ± 1.53	4.75 ± 1.89	QHD + CT	CT	28 d	⑥⑦⑫
Liu L et al. (2023)	59	55	37/22	35/20	62.7 ± 5.9	62.5 ± 6.3	7.86 ± 2.17	7.54 ± 2.11	QHD + CT	CT	14 d	③⑥⑦⑫⑬
Ni (2021)	35	35	18/17	20/15	60.2 ± 3.3	61.2 ± 3.8	5.37 ± 1.03	5.33 ± 1.02	QHD + CT	CT	14 d	①②③⑥⑦⑨⑩
Qin. (2022)	65	65	33/32	35/30	64.92 ± 11.45	65.48 ± 12.17	—	—	QHD + CT	CT	14 d	①②③⑫
Sun. (2020)	39	39	21/18	22/17	72.30 ± 5.16	71.10 ± 5.62	3.85 ± 0.82	3.89 ± 0.78	QHD + CT	CT	10 d	⑥⑦
Tang et al. (2020)	93	93	59/34	56/37	58.69 ± 7.83	58.27 ± 7.39	13.17 ± 2.38	12.52 ± 2.14	QHD + CT	CT	14 d	①②③④⑤⑥⑨⑪⑫⑬
Wang (2019)	46	46	25/21	26/20	55.97 ± 6.94	56.34 ± 6.37	8.04 ± 2.97	7.96 ± 2.36	QHD + CT	CT	14 d	①②③⑫⑬
Wang (2022)	35	35	26/9	24/11	65.58 ± 5.23	65.31 ± 5.74	7.66 ± 1.65	7.27 ± 1.61	QHD + CT	CT	14 d	①②④⑤⑫⑬
Wei (2020)	31	31	13/18	10/21	55.03 ± 5.41	56.06 ± 6.22	10.16 ± 0.73	10.90 ± 0.89	QHD + CT	CT	14 d	①③⑫
Wei and Niu (2017)	30	30	18/12	19/11	52–72	51–73	2–10	2–11	QHD + CT	CT	7 d	⑩⑪⑫
Wen (2022)	44	44	23/21	24/20	64.97 ± 3.82	65.02 ± 3.04	—	—	QHD + CT	CT	15 d	①②③⑫⑬
Wu. (2014)	35	35	19/16	17/18	64.12 ± 9.07	63.58 ± 8.74	15.93 ± 10.16	16.24 ± 11.75	QHD + CT	CT	10 d	①②③⑥⑨⑩⑫
Xie (2016)	30	30	11/19	13/17	57.2 ± 8.2	56.7 ± 7.5	6.7 ± 2.4	6.9 ± 2.2	QHD + CT	CT	14 d	①②③⑨
Yang et al. (2023b)	42	42	28/14	30/12	82.98 ± 8.26	80.26 ± 6.62	—	—	QHD + CT	CT	14 d	⑩⑪⑫
Yu et al. (2022)	67	68	42/25	44/24	71.2 ± 12.1	72.5 ± 11.6	6.5 ± 1.4	6.6 ± 1.3	QHD + CT	CT	14 d	⑩⑪⑫
Yu (2019)	55	55	31/24	29/26	62.8 ± 3.1	63.2 ± 2.4	—	—	QHD + CT	CT	7 d	①②③⑧⑩⑪⑫⑬
Yuan (2018)	50	50	30/20	29/21	52.17 ± 6.33	52.20 ± 6.37	5.25 ± 1.41	5.27 ± 1.38	QHD + CT	CT	10 d	①②③④⑫
Zhang B. R (2018)	30	30	15/15	15/15	57.28 ± 4.26	57.34 ± 4.31	—	—	QHD + CT	CT	10 d	①②③⑫
Zhang F (2018)	43	43	25/18	23/20	68.35 ± 7.49	69.12 ± 8.56	5.32 ± 1.38	4.98 ± 1.43	QHD + CT	CT	14 d	②③⑥⑧⑩⑪⑫
Zhang G et al. (2021)	45	45	26/19	27/18	61.88 ± 5.44	62.21 ± 5.37	4.73 ± 1.16	4.65 ± 1.21	QHD + CT	CT	10 d	①②③④⑤⑥⑨
Zhang and Li (2020)	30	30	17/13	18/12	55.0 ± 1.5	55.6 ± 1.4	—	—	QHD + CT	CT	10 d	①②③⑫
Zhang and Ge (2022)	40	40	35/5	33/7	39	39.6	10.74 ± 1.49	10.88 ± 1.60	QHD + CT	CT	14 d	②⑥⑧⑩⑫
Zhang et al. (2016)	60	57	34/26	34/23	63.50 ± 3.26	63.04 ± 3.28	5.62 ± 2.72	5.50 ± 2.68	QHD + CT	CT	7 d	⑥⑨⑫⑬
Zhang et al. (2015)	50	50	54/46		60.5 ± 4.2		—	—	QHD + CT	CT	10 d	①②③④⑤⑥⑨⑩⑫
Zhao (2016)	30	30	19/11	20/10	50–70	50–70	0.5–11	1–11	QHD + CT	CT	14 d	①②③④⑤⑧⑨⑩⑪⑫
Zhao (2023)	30	30	17/13	16/14	58.25 ± 3.39	58.27 ± 3.41	6.71 ± 0.83	6.73 ± 0.82	QHD + CT	CT	14 d	①②⑦⑫
Zhou et al. (2014)	45	45	58/32		50–71		—	—	QHD + CT	CT	14 d	①②③④⑤⑫
Zhou et al. (2016)	50	50	27/23	29/21	66.2 ± 9.2	62.4 ± 9.5	13.1 ± 5.3	12.5 ± 5.2	QHD + CT	CT	14 d	①②③④⑤⑫
Zhu (2021)	29	29	15/14	16/13	68.79 ± 5.29	67.83 ± 5.46	4.92 ± 1.84	4.55 ± 1.79	QHD + CT	CT	7 d	②③⑥⑦⑫

Note: C, control group; T, treatment group; M, male; F, female; COPD, chronic obstructive pulmonary disease; QHD, *qingjin huatan* decoction; CT, conventional treatment; Outcomes: ①FEV1; ②FVC; ③FEV1/FVC; ④PaO_2_; ⑤PaCO_2_; ⑥TNF-α; ⑦IL-1β; ⑧IL-6; ⑨IL-8; ⑩CRP; ⑪PCT; ⑫Clinical efficacy; ⑬Adverse reactions. -, not reported.

### 3.2 Risk of bias assessment

**FIGURE 2:**
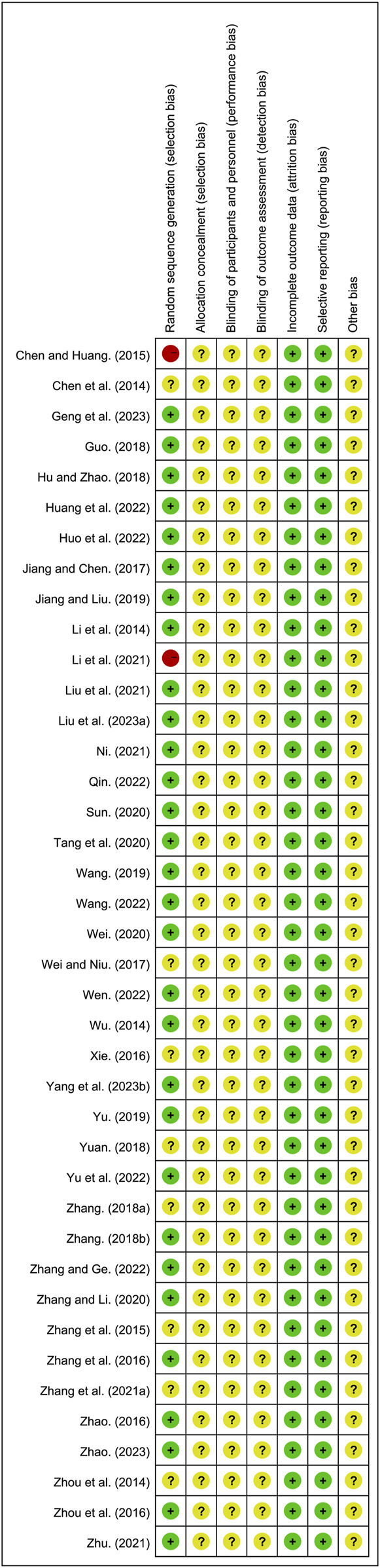
Bias risk assessment of included studies.

All included studies were RCTs. Of these, 30 studies (Geng et al., 2023; Guo, 2018; Hu and Zhao, 2018; Huang et al., 2022; Huo et al., 2022; Jiang and Liu, 2019; Jiang and Chen, 2017; Li et al., 2014; Liu et al., 2021; Liu L. et al., 2023; Ni, 2021; Qin, 2022; Sun, 2020; Tang et al., 2020; Wang, 2019; Wang, 2022; Wei, 2020; Wen, 2022; Wu, 2014; Yang et al., 2023b; Yu et al., 2022; Yu, 2019; Zhang F., 2018; Zhang G. et al., 2021; Zhang and Ge, 2022; Zhang et al., 2016; Zhao, 2016; Zhao, 2023; Zhou et al., 2016; Zhu, 2021) that utilized random number table methods and were designated as low risk. However, 8 studies (Chen et al., 2014; Wei and Niu, 2017; Xie, 2016; Yuan, 2018; Zhang B. R., 2018; Zhang and Li, 2020; Zhang et al., 2015; Zhou et al., 2014) did not provide specific randomization methods and were therefore classified as unclear risk. One study (Li et al., 2021) grouped participants based on medication usage, and another (Chen and Huang, 2015) based on hospital admission numbers, both of which were designated as high risk. None of the included studies reported on allocation concealment and blinding, leading to their classification as unclear risk. All studies reported complete outcome and were designated as low risk. The risk assessment of bias is shown in Figure 2 and Supplementary Material S4.

### 3.3 Primary outcomes

#### 3.3.1 FEV1

**FIGURE 3:**
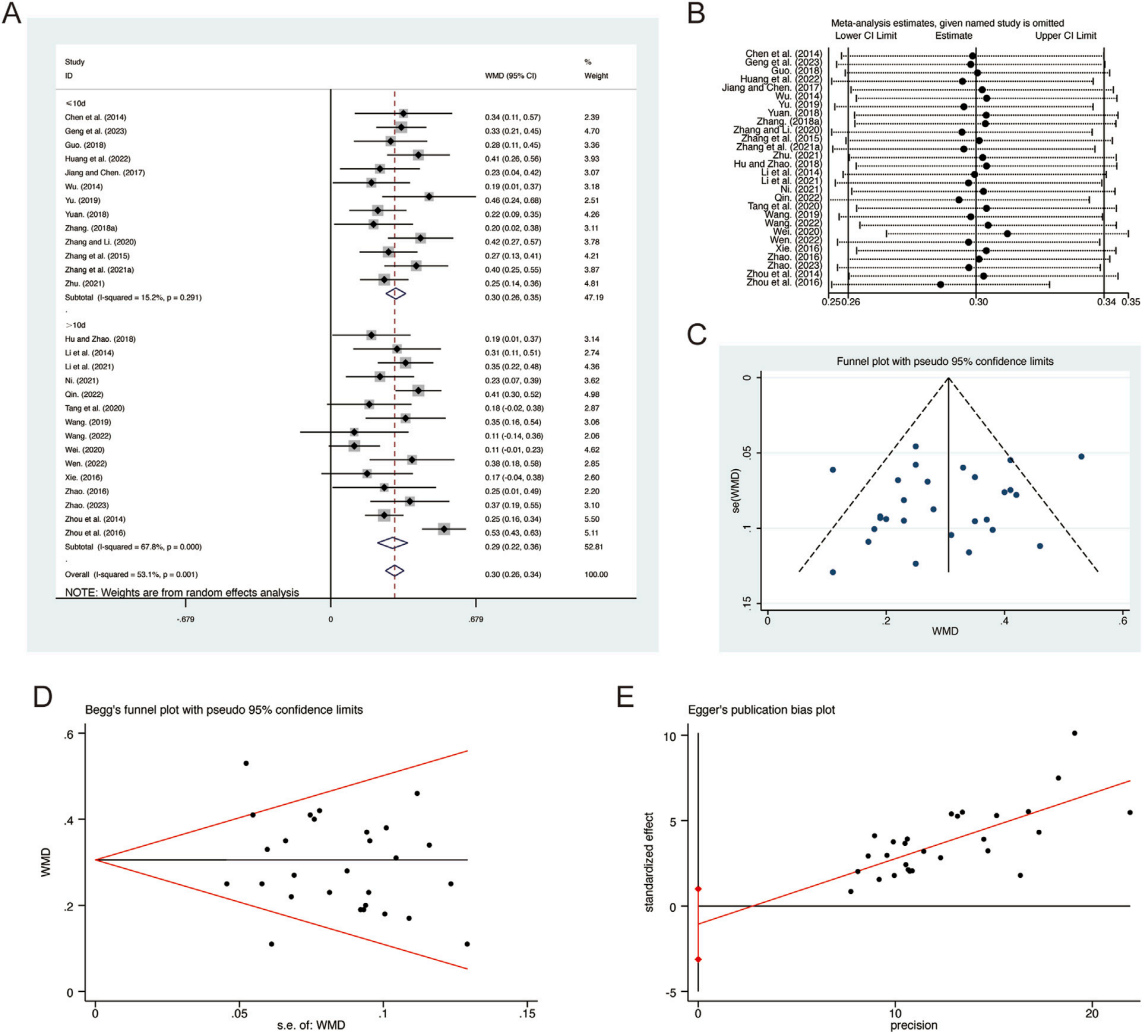
Effect of QHD on FEV1 in patients with AECOPD. **(A)** Forest plot for FEV1. **(B)** Sensitivity analysis for FEV1. **(C)** Funnel plot for FEV1. **(D)** Begg’s test for FEV1. **(E)** Egger’s test for FEV1.

A total of 28 studies (Chen et al., 2014; Geng et al., 2023; Guo, 2018; Hu and Zhao, 2018; Huang et al., 2022; Jiang and Chen, 2017; Li et al., 2014; Li et al., 2021; Ni, 2021; Qin, 2022; Tang et al., 2020; Wang, 2019; Wang, 2022; Wei, 2020; Wen, 2022; Wu, 2014; Xie, 2016; Yu, 2019; Yuan, 2018; Zhang B. R., 2018; Zhang G. et al., 2021; Zhang and Li, 2020; Zhang et al., 2015; Zhao, 2016; Zhao, 2023; Zhou et al., 2014; Zhou et al., 2016; Zhu, 2021) reported on FEV1, demonstrating significant heterogeneity among the studies (*I* ^*2*^ = 53.1%, *p* = 0.001). Consequently, a random-effects model was employed to pool the effect sizes. The analysis revealed that, compared to CT, QHD significantly improved FEV1 (MD = 0.30, 95% CI: 0.26 to 0.34, *p* = 0.000, Figure 3A). Subgroup analysis based on the duration of QHD treatment indicated significant differences between QHD and CT for both treatment durations: less than 10 days (MD = 0.30, 95% CI: 0.26 to 0.35, *p* = 0.000, Figure 3A) and more than 10 days (MD = 0.29, 95% CI: 0.22 to 0.36, *p* = 0.000, Figure 3A). Sensitivity analysis supported the robustness and reliability of these pooled results (Figure 3B). Furthermore, no publication bias was detected as evidenced by Begg’s test (*p* = 0.594) and Egger’s test (*p* = 0.302) (Figures 3C–E).

#### 3.3.2 FVC

**FIGURE 4:**
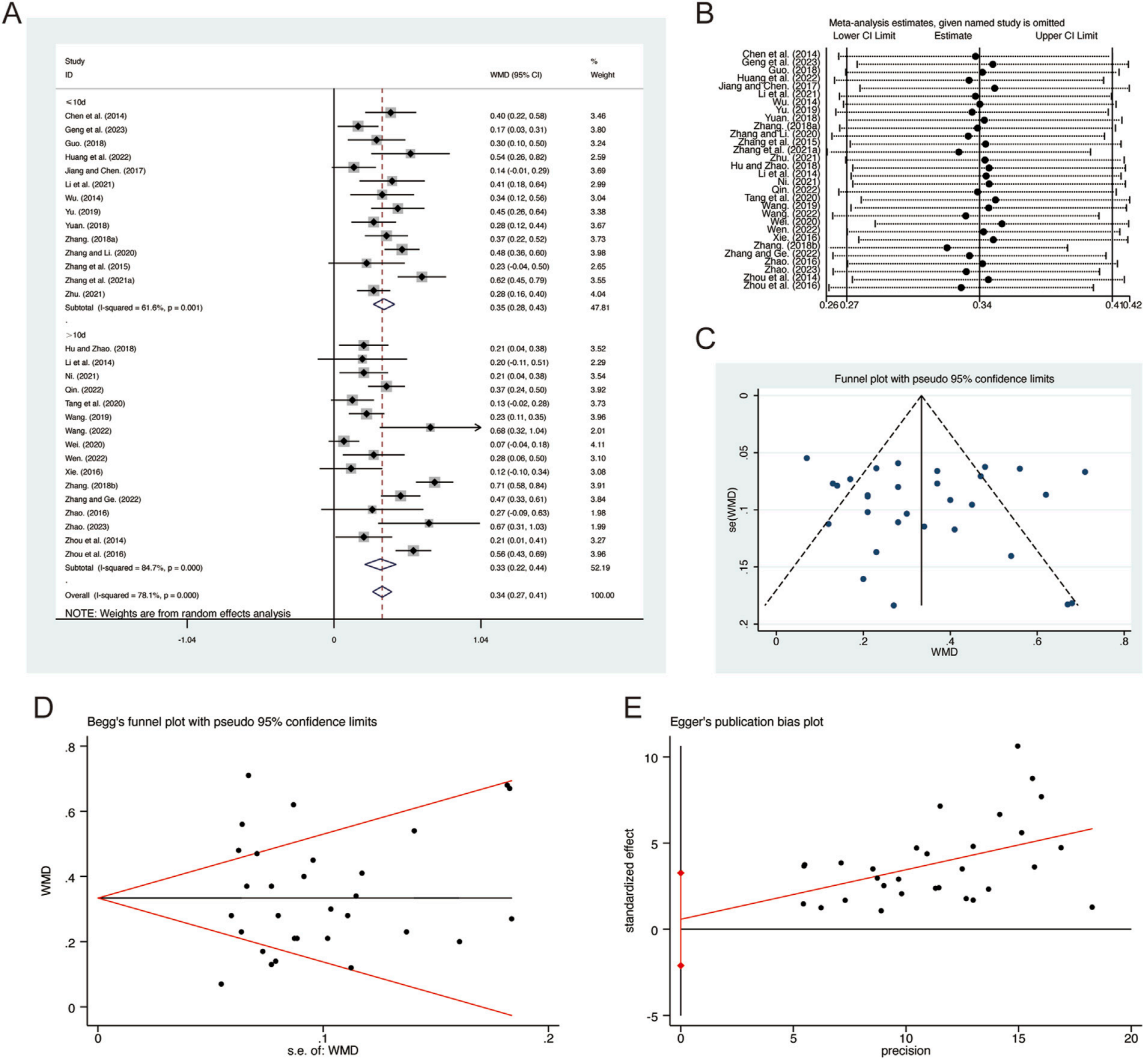
Effect of QHD on FVC in patients with AECOPD. **(A)** Forest plot for FVC. **(B)** Sensitivity analysis for FVC. **(C)** Funnel plot for FVC. **(D)** Begg’s test for FVC. **(E)** Egger’s test for FVC.

A total of 30 studies (Chen et al., 2014; Geng et al., 2023; Guo, 2018; Hu and Zhao, 2018; Huang et al., 2022; Jiang and Chen, 2017; Li et al., 2014; Li et al., 2021; Ni, 2021; Qin, 2022; Tang et al., 2020; Wang, 2019; Wang, 2022; Wei and Niu, 2017; Wen, 2022; Wu, 2014; Xie, 2016; Yu, 2019; Yuan, 2018; Zhang B. R., 2018; Zhang F., 2018; Zhang G. et al., 2021; Zhang and Li, 2020; Zhang and Ge, 2022; Zhang et al., 2015; Zhao, 2016; Zhao, 2023; Zhou et al., 2014; Zhou et al., 2016; Zhu, 2021) reported on FVC, demonstrating significant heterogeneity among the studies (*I* ^*2*^ = 78.1%, *p* = 0.001). Consequently, a random-effects model was employed to pool the effect sizes. The analysis revealed that, compared to CT, QHD significantly improved FVC (MD = 0.34, 95% CI: 0.27 to 0.41, *p* = 0.000, Figure 4A). Subgroup analysis based on the duration of QHD treatment indicated significant differences between QHD and CT for both treatment durations: less than 10 days (MD = 0.35, 95% CI: 0.28 to 0.43, *p* = 0.000, Figure 4A) and more than 10 days (MD = 0.33, 95% CI: 0.22 to 0.44, *p* = 0.000, Figure 4A). Sensitivity analysis supported the robustness and reliability of these pooled results (Figure 4B). Furthermore, no publication bias was detected as evidenced by Begg’s test (*p* = 0.454) and Egger’s test (*p* = 0.664) (Figures 4C–E).

#### 3.3.3 FEV1/FVC

**FIGURE 5:**
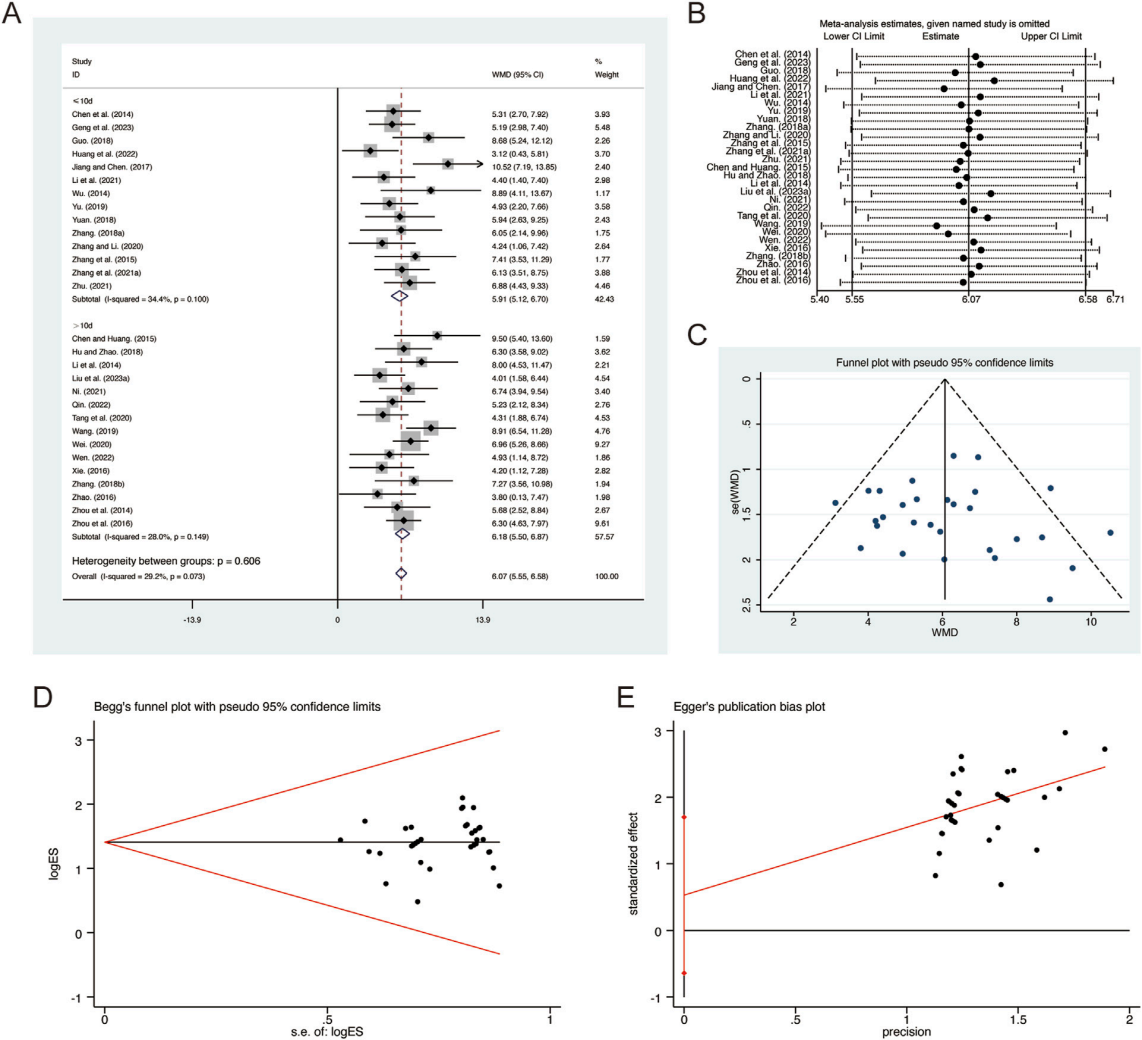
Effect of QHD on FEV1/FVC in patients with AECOPD. **(A)** Forest plot for FEV1/FVC. **(B)** Sensitivity analysis for FEV1/FVC. **(C)** Funnel plot for FEV1/FVC. **(D)** Begg’s test for FEV1/FVC. **(E)** Egger’s test for FEV1/FVC.

A total of 29 studies (Chen et al., 2014; Chen and Huang, 2015; Geng et al., 2023; Guo, 2018; Hu and Zhao, 2018; Huang et al., 2022; Jiang and Chen, 2017; Li et al., 2014; Li et al., 2021; Liu L. et al., 2023; Ni, 2021; Qin, 2022; Tang et al., 2020; Wang, 2019; Wei, 2020; Wen, 2022; Wu, 2014; Xie, 2016; Yu, 2019; Yuan, 2018; Zhang B. R., 2018; Zhang F., 2018; Zhang G. et al., 2021; Zhang and Li, 2020; Zhang et al., 2015; Zhao, 2016; Zhou et al., 2014; Zhou et al., 2016; Zhu, 2021) reported on FEV1/FVC, demonstrating no significant heterogeneity among the studies (*I* ^*2*^ = 29.2%, *p* = 0.073). Consequently, a fixed-effects model was employed to pool the effect sizes. The analysis revealed that, compared to CT, QHD significantly improved FEV1/FVC (MD = 6.07, 95% CI: 5.55 to 6.58, *p* = 0.000, Figure 5A). Subgroup analysis based on the duration of QHD treatment indicated significant differences between QHD and CT for both treatment durations: less than 10 days (MD = 5.91, 95% CI: 5.12 to 6.70, *p* = 0.000, Figure 5A) and more than 10 days (MD = 6.18, 95% CI: 5.50 to 6.87, *p* = 0.000, Figure 5A). Sensitivity analysis supported the robustness and reliability of these pooled results (Figure 5B). Furthermore, no publication bias was detected as evidenced by Begg’s test (*p* = 0.183) and Egger’s test (*p* = 0.604) (Figures 5C–E).

#### 3.3.4 PaO_2_

**FIGURE 6:**
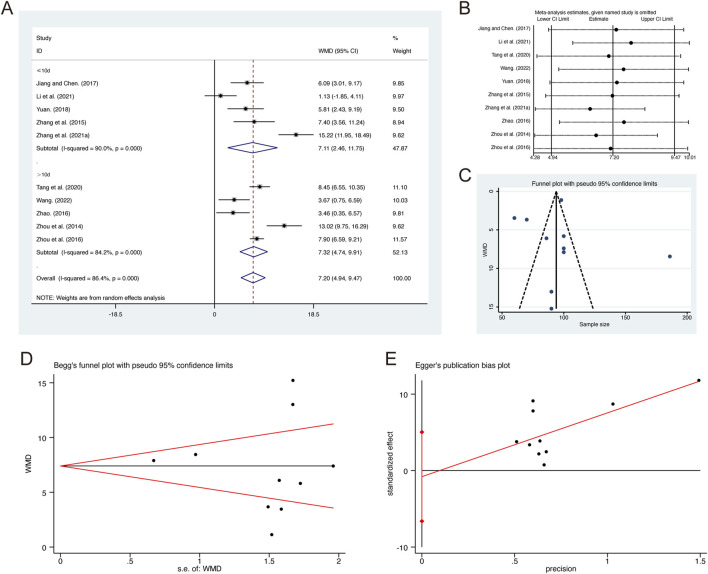
Effect of QHD on PaO_2_ in patients with AECOPD. **(A)** Forest plot for PaO_2_. **(B)** Sensitivity analysis for PaO_2_. **(C)** Funnel plot for PaO_2_. **(D)** Begg’s test for PaO_2_. **(E)** Egger’s test for PaO_2_.

A total of 10 studies (Jiang and Chen, 2017; Li et al., 2021; Tang et al., 2020; Wang, 2022; Yuan, 2018; Zhang G. et al., 2021; Zhang et al., 2015; Zhao, 2016; Zhou et al., 2014; Zhou et al., 2016) reported on PaO2, demonstrating significant heterogeneity among the studies (*I* ^*2*^ = 86.4%, *p* = 0.000). Consequently, a random-effects model was employed to pool the effect sizes. The analysis revealed that, compared to CT, QHD significantly improved PaO2 (MD = 7.20, 95% CI: 4.94 to 9.47, *p* = 0.000, Figure 6A). Subgroup analysis based on the duration of QHD treatment indicated significant differences between QHD and CT for both treatment durations: less than 10 days (MD = 7.11, 95% CI: 2.46 to 11.75, *p* = 0.000, Figure 6A) and more than 10 days (MD = 7.32, 95% CI: 4.74 to 9.91, *p* = 0.000, Figure 6A). Sensitivity analysis supported the robustness and reliability of these pooled results (Figure 6B). Furthermore, no publication bias was detected as evidenced by Begg’s test (*p* = 0.531) and Egger’s test (*p* = 0.761) (Figures 6C–E).

#### 3.3.5 PaCO_2_

**FIGURE 7:**
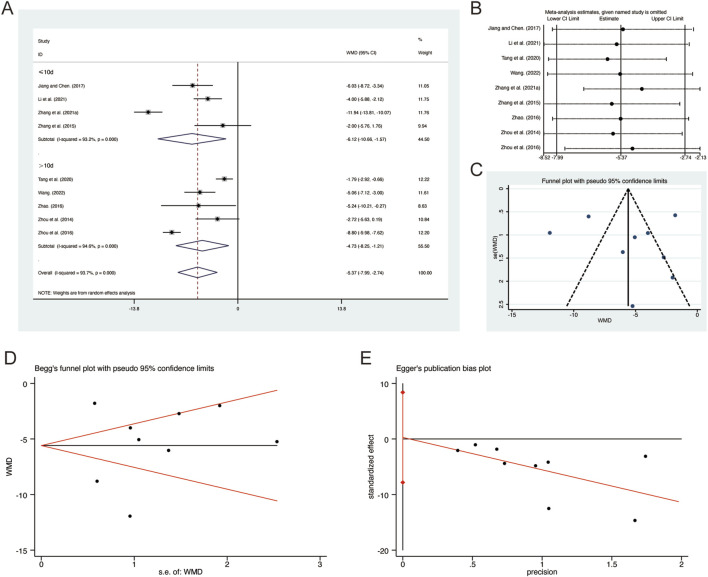
Effect of QHD on PaCO_2_ in patients with AECOPD. **(A)** Forest plot for PaCO_2_. **(B)** Sensitivity analysis for PaCO_2_. **(C)** Funnel plot for PaCO_2_. **(D)** Begg’s test for PaCO_2_. **(E)** Egger’s test for PaCO_2_.

A total of 9 studies (Jiang and Chen, 2017; Li et al., 2021; Tang et al., 2020; Wang, 2022; Zhang G. et al., 2021; Zhang et al., 2015; Zhao, 2016; Zhou et al., 2014; Zhou et al., 2016) reported on PaCO2, demonstrating significant heterogeneity among the studies (*I* ^*2*^ = 93.7%, *p* = 0.000). Consequently, a random-effects model was employed to pool the effect sizes. The analysis revealed that, compared to CT, QHD significantly reduced PaCO2 (MD = −5.37, 95% CI: −7.99 to −2.74, *p* = 0.000, Figure 7A). Subgroup analysis based on the duration of QHD treatment indicated significant differences between QHD and CT for both treatment durations: less than 10 days (MD = −5.12, 95% CI: −10.66 to −1.57, *p* = 0.000, Figure 7A) and more than 10 days (MD = −4.73, 95% CI: −8.25 to −1.21, *p* = 0.000, Figure 7A). Sensitivity analysis supported the robustness and reliability of these pooled results (Figure 7B). Furthermore, no publication bias was detected as evidenced by Begg’s test (*p* = 0.835) and Egger’s test (*p* = 0.936) (Figures 7C–E).

#### 3.3.6 TNF-α

**FIGURE 8:**
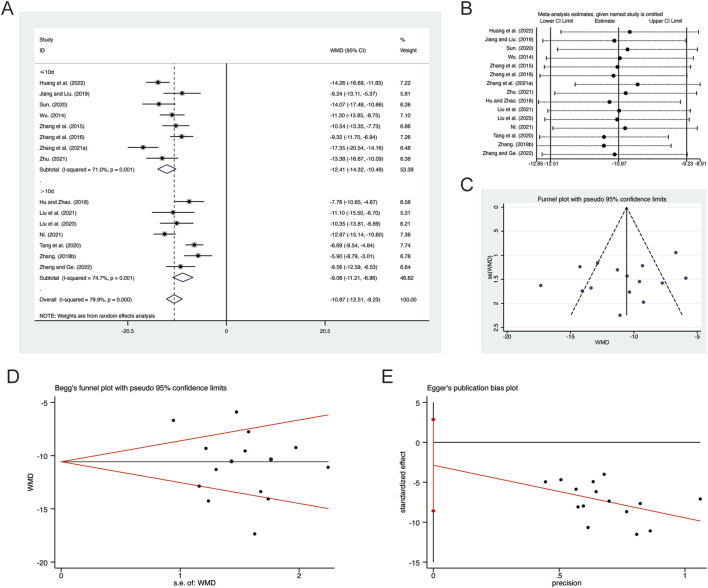
Effect of QHD on TNF-α in patients with AECOPD. **(A)** Forest plot for TNF-α. **(B)** Sensitivity analysis for TNF-α. **(C)** Funnel plot for TNF-α. **(D)** Begg’s test for TNF-α. **(E)** Egger’s test for TNF-α.

A total of 15 studies (Hu and Zhao, 2018; Huang et al., 2022; Jiang and Liu, 2019; Liu et al., 2021; Liu L. et al., 2023; Ni, 2021; Sun, 2020; Tang et al., 2020; Wu, 2014; Zhang F., 2018; Zhang G. et al., 2021; Zhang and Ge, 2022; Zhang et al., 2016; Zhang et al., 2015; Zhu, 2021) reported on TNF-α, demonstrating significant heterogeneity among the studies (*I* ^*2*^ = 79.9%, *p* = 0.000). Consequently, a random-effects model was employed to pool the effect sizes. The analysis revealed that, compared to CT, QHD significantly reduced TNF-α (MD = −10.87, 95% CI: −12.51 to −9.23, *p* = 0.000, Figure 8A). Subgroup analysis based on the duration of QHD treatment indicated significant differences between QHD and CT for both treatment durations: less than 10 days (MD = −12.41, 95% CI: −14.32 to −10.49, *p* = 0.000, Figure 8A) and more than 10 days (MD = −9.08, 95% CI: −11.21 to −6.96, *p* = 0.000, Figure 8A). Sensitivity analysis supported the robustness and reliability of these pooled results (Figure 8B). Furthermore, no publication bias was detected as evidenced by Begg’s test (*p* = 0.767) and Egger’s test (*p* = 0.298) (Figures 8C–E).

#### 3.3.7 IL-1β

**FIGURE 9:**
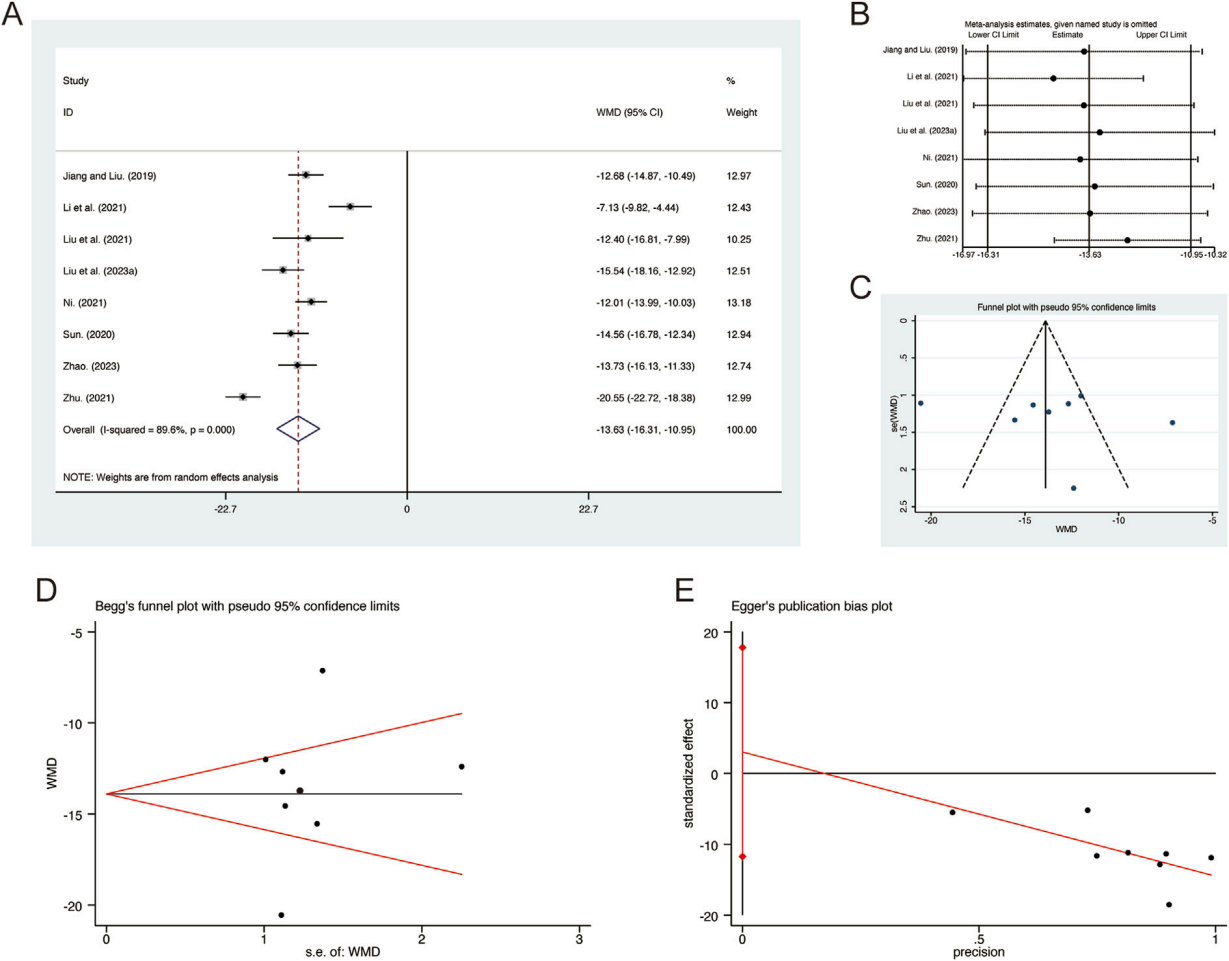
Effect of QHD on IL-1β in patients with AECOPD. **(A)** Forest plot for IL-1β. **(B)** Sensitivity analysis for IL-1β. **(C)** Funnel plot for IL-1β. **(D)** Begg’s test for IL-1β. **(E)** Egger’s test for IL-1β.

A total of 8 studies (Jiang and Liu, 2019; Li et al., 2021; Liu et al., 2021; Liu L. et al., 2023; Ni, 2021; Sun, 2020; Zhao, 2023; Zhu, 2021) reported on IL-1β, demonstrating significant heterogeneity among the studies (*I* ^*2*^ = 89.6%, *p* = 0.000). Consequently, a random-effects model was employed to pool the effect sizes. The analysis revealed that, compared to CT, QHD significantly reduced IL-1β (MD = −13.63, 95% CI: −16.31 to −10.95, *p* = 0.000, Figure 9A). Sensitivity analysis supported the robustness and reliability of these pooled results (Figure 9B). Furthermore, no publication bias was detected as evidenced by Begg’s test (*p* = 0.902) and Egger’s test (*p* = 0.634) (Figures 9C–E).

#### 3.3.8 IL-6

**FIGURE 10:**
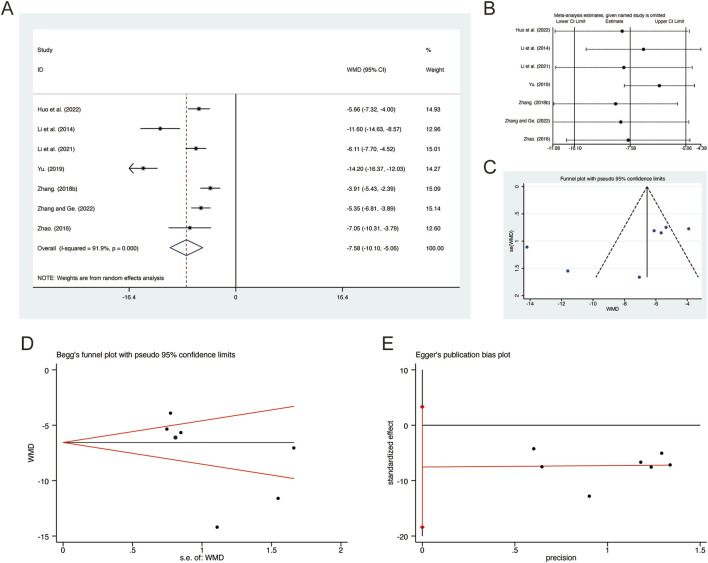
Effect of QHD on IL-6 in patients with AECOPD. **(A)** Forest plot for IL-6. **(B)** Sensitivity analysis for IL-6. **(C)** Funnel plot for IL-6. **(D)** Begg’s test for IL-6. **(E)** Egger’s test for IL-6.

A total of 7 studies (Huo et al., 2022; Li et al., 2014; Li et al., 2021; Yu, 2019; Zhang F., 2018; Zhang and Ge, 2022; Zhao, 2016) reported on IL-6, demonstrating significant heterogeneity among the studies (*I* ^*2*^ = 91.9%, *p* = 0.000). Consequently, a random-effects model was employed to pool the effect sizes. The analysis revealed that, compared to CT, QHD significantly reduced IL-6 (MD = −7.58, 95% CI: −10.10 to −5.06, *p* = 0.000, Figure 10A). Sensitivity analysis supported the robustness and reliability of these pooled results (Figure 10B). Furthermore, no publication bias was detected as evidenced by Begg’s test (*p* = 0.133) and Egger’s test (*p* = 0.134) (Figures 10C–E).

#### 3.3.9 IL-8

**FIGURE 11:**
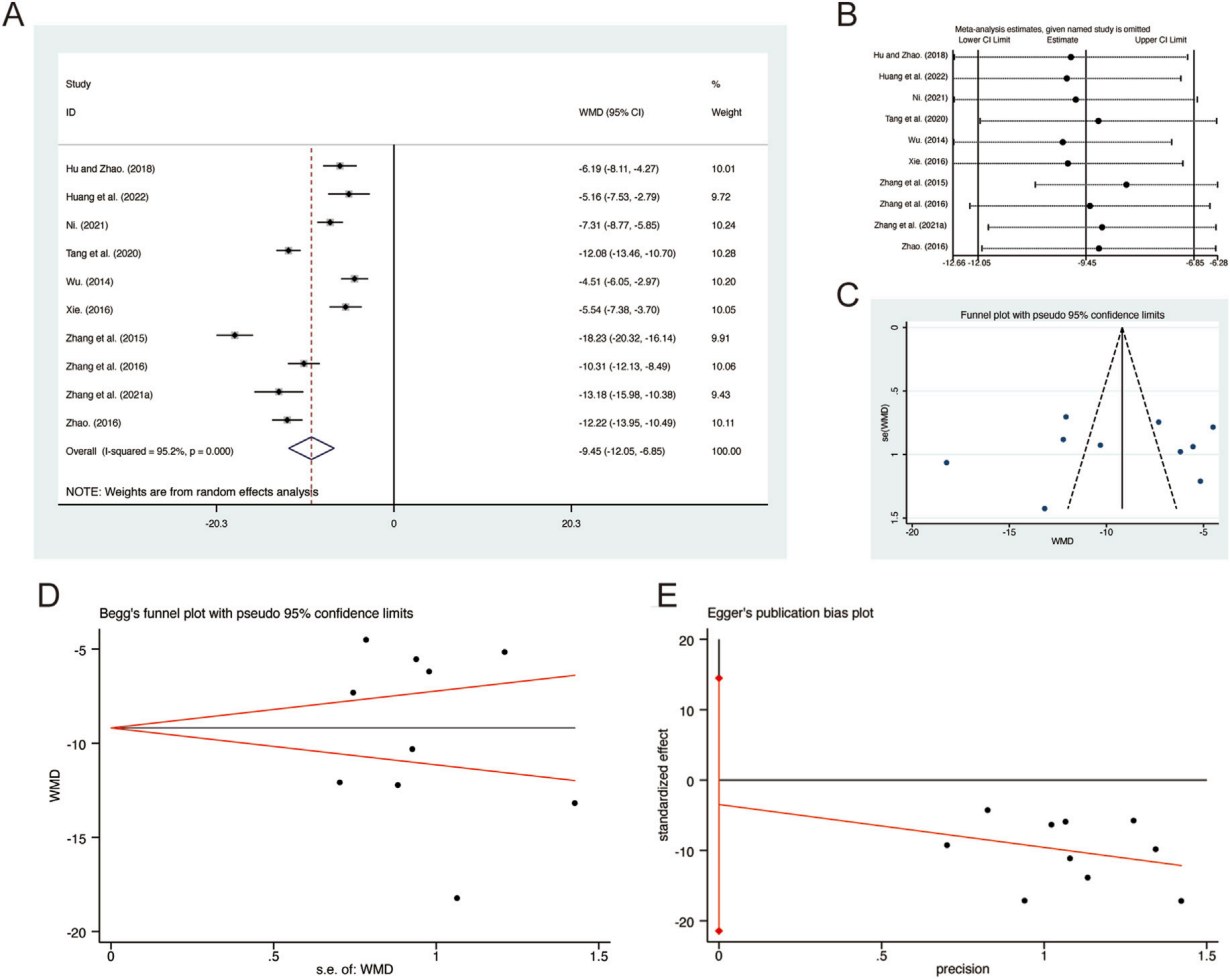
Effect of QHD on IL-8 in patients with AECOPD. **(A)** Forest plot for IL-8. **(B)** Sensitivity analysis for IL-8. **(C)** Funnel plot for IL-8. **(D)** Begg’s test for IL-8. **(E)** Egger’s test for IL-8.

A total of 10 studies (Hu and Zhao, 2018; Huang et al., 2022; Ni, 2021; Tang et al., 2020; Wu, 2014; Xie, 2016; Zhang G. et al., 2021; Zhang et al., 2016; Zhang et al., 2015; Zhao, 2016) reported on IL-8, demonstrating significant heterogeneity among the studies (*I* ^*2*^ = 95.2%, *p* = 0.000). Consequently, a random-effects model was employed to pool the effect sizes. The analysis revealed that, compared to CT, QHD significantly reduced IL-8 (MD = −9.45, 95% CI: −12.05 to −6.85, *p* = 0.000, Figure 11A). Sensitivity analysis supported the robustness and reliability of these pooled results (Figure 11B). Furthermore, no publication bias was detected as evidenced by Begg’s test (*p* = 1.000) and Egger’s test (*p* = 0.668) (Figures 11C–E).

#### 3.3.10 CRP

**FIGURE 12:**
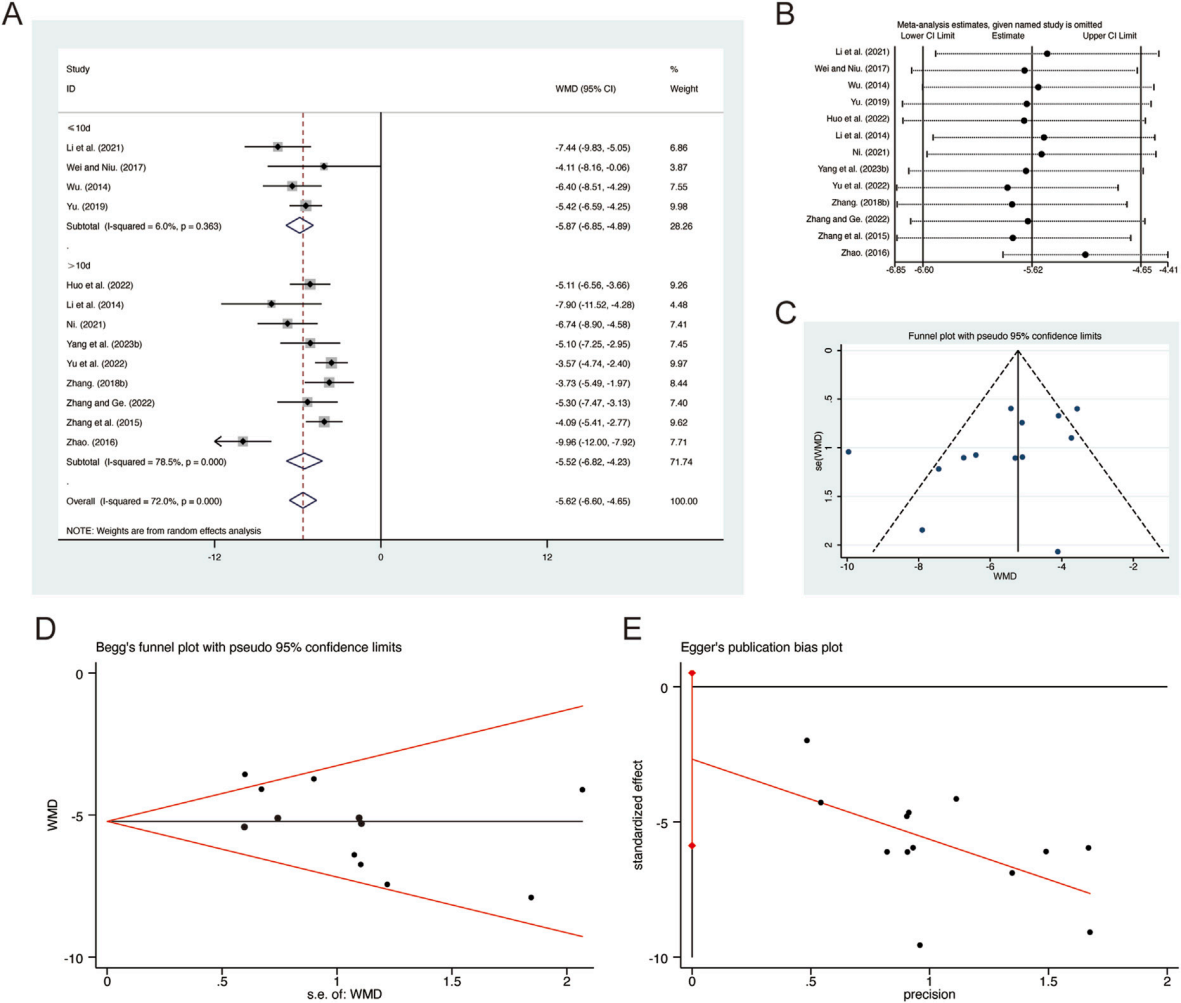
Effect of QHD on CRP in patients with AECOPD. **(A)** Forest plot for CRP. **(B)** Sensitivity analysis for CRP. **(C)** Funnel plot for CRP. **(D)** Begg’s test for CRP. **(E)** Egger’s test for CRP.

A total of 13 studies (Huo et al., 2022; Li et al., 2014; Li et al., 2021; Ni, 2021; Wei and Niu, 2017; Wu, 2014; Yang et al., 2023b; Yu et al., 2022; Yu, 2019; Zhang F., 2018; Zhang and Ge, 2022; Zhang et al., 2015; Zhao, 2016) reported on CRP, demonstrating significant heterogeneity among the studies (*I* ^*2*^ = 72.0%, *p* = 0.000). Consequently, a random-effects model was employed to pool the effect sizes. The analysis revealed that, compared to CT, QHD significantly reduced CRP (MD = −5.62, 95% CI: −6.60 to −4.65, *p* = 0.000, Figure 12A). Subgroup analysis based on the duration of QHD treatment indicated significant differences between QHD and CT for both treatment durations: less than 10 days (MD = −5.87, 95% CI: −6.85 to −4.89, *p* = 0.000, Figure 12A) and more than 10 days (MD = −5.52, 95% CI: −6.82 to −4.23, *p* = 0.000, Figure 12A). Sensitivity analysis supported the robustness and reliability of these pooled results (Figure 12B). Furthermore, no publication bias was detected as evidenced by Begg’s test (*p* = 0.127) and Egger’s test (*p* = 0.092) (Figures 12C–E).

#### 3.3.11 PCT

**FIGURE 13:**
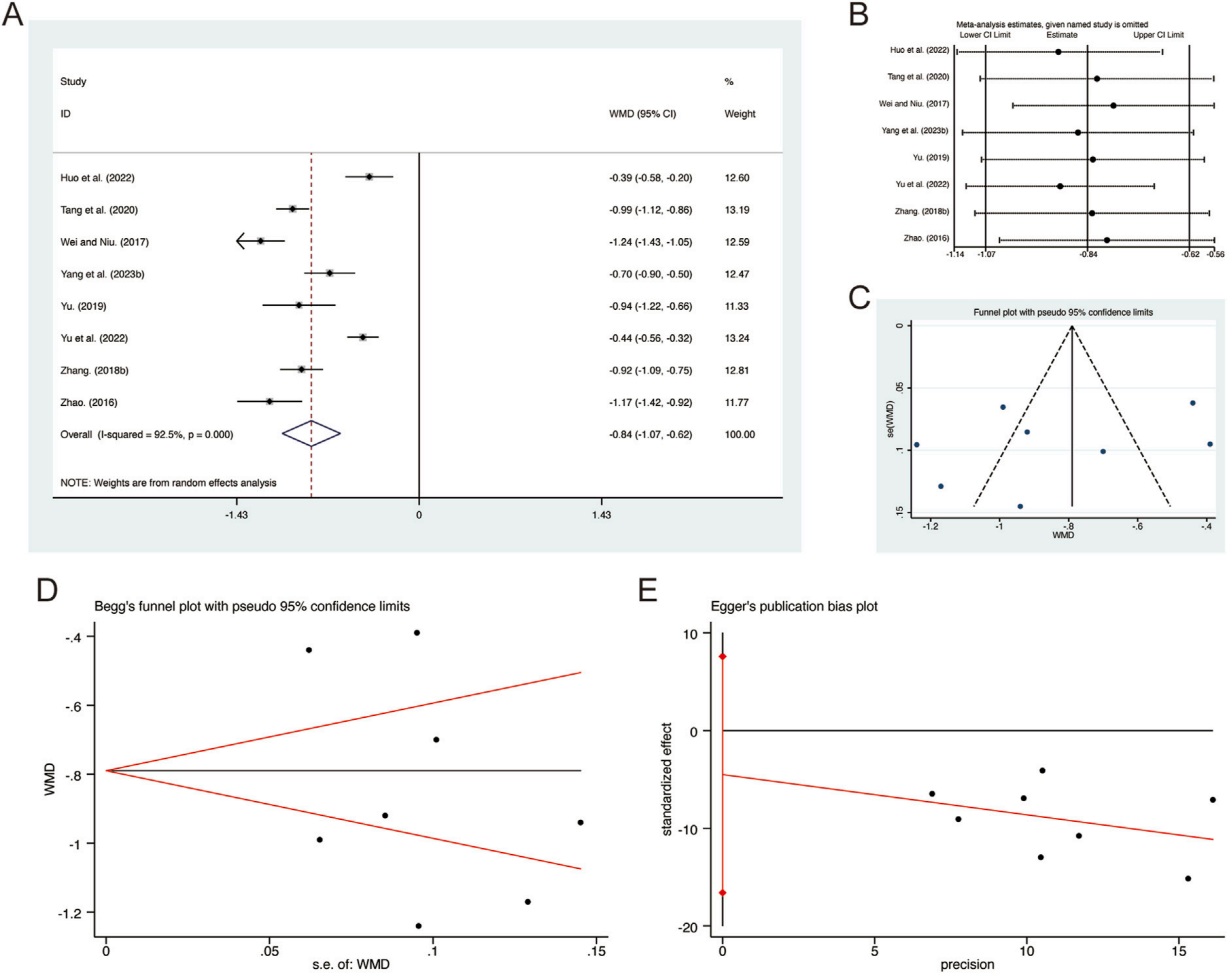
Effect of QHD on PCT in patients with AECOPD. **(A)** Forest plot for PCT. **(B)** Sensitivity analysis for PCT. **(C)** Funnel plot for PCT. **(D)** Begg’s test for PCT. **(E)** Egger’s test for PCT.

A total of 8 studies (Huo et al., 2022; Tang et al., 2020; Wei and Niu, 2017; Yang et al., 2023b; Yu et al., 2022; Yu, 2019; Zhang F., 2018; Zhao, 2016) reported on PCT, demonstrating significant heterogeneity among the studies (*I* ^*2*^ = 92.5%, *p* = 0.000). Consequently, a random-effects model was employed to pool the effect sizes. The analysis revealed that, compared to CT, QHD significantly reduced PCT (MD = −0.84, 95% CI: −1.07 to −0.62, *p* = 0.000, Figure 13A). Sensitivity analysis supported the robustness and reliability of these pooled results (Figure 13B). Furthermore, no publication bias was detected as evidenced by Begg’s test (*p* = 0.711) and Egger’s test (*p* = 0.396) (Figures 13C–E).

### 3.4 Secondary outcomes

#### 3.4.1 Clinical efficacy

**FIGURE 14:**
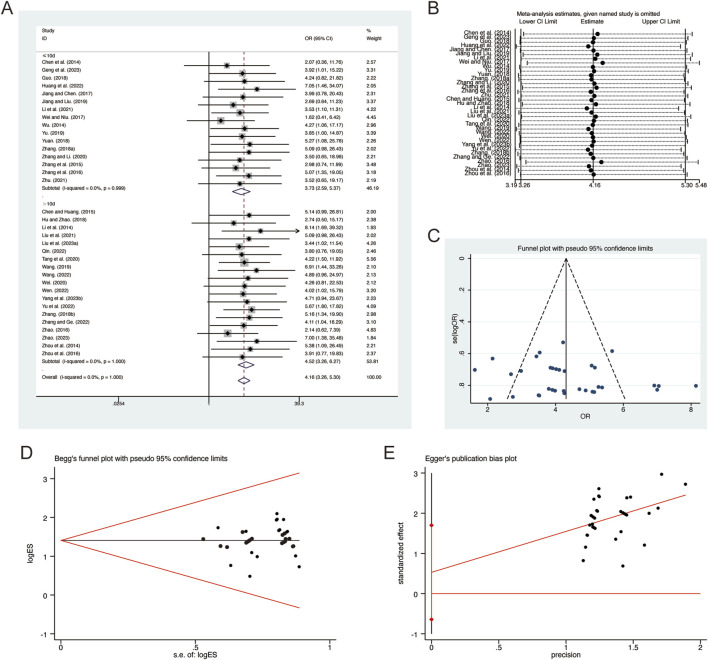
Effect of QHD on clinical efficacy in patients with AECOPD. **(A)** Forest plot for clinical efficacy. **(B)** Sensitivity analysis for clinical efficacy. **(C)** Funnel plot for clinical efficacy. **(D)** Begg’s test for clinical efficacy. **(E)** Egger’s test for clinical efficacy.

A total of 35 studies (Chen et al., 2014; Chen and Huang, 2015; Geng et al., 2023; Guo, 2018; Hu and Zhao, 2018; Huang et al., 2022; Jiang and Liu, 2019; Jiang and Chen, 2017; Li et al., 2014; Li et al., 2021; Liu et al., 2021; Liu L. et al., 2023; Qin, 2022; Tang et al., 2020; Wang, 2019; Wang, 2022; Wei, 2020; Wei and Niu, 2017; Wen, 2022; Yang et al., 2023b; Yu et al., 2022; Yu, 2019; Yuan, 2018; Zhang B. R., 2018; Zhang F., 2018; Zhang and Li, 2020; Zhang and Ge, 2022; Zhang et al., 2016; Zhang et al., 2015; Zhao, 2016; Zhao, 2023; Zhou et al., 2014; Zhou et al., 2016; Zhu, 2021) reported on clinical efficacy, demonstrating no significant heterogeneity among the studies (*I* ^*2*^ = 0.0%, *p* = 1.000). Consequently, a fixed-effects model was employed to pool the effect sizes. The analysis revealed that, compared to CT, QHD significantly improved clinical efficacy (RR = 4.16, 95% CI: 3.26 to 5.30, *p* = 0.000, Figure 14A). Subgroup analysis based on the duration of QHD treatment indicated significant differences between QHD and CT for both treatment durations: less than 10 days (RR = 3.73, 95% CI: 2.59 to 5.37, *p* = 0.000, Figure 14A) and more than 10 days (RR = 4.52, 95% CI: 3.26 to 6.27, *p* = 0.000, Figure 14A). Sensitivity analysis supported the robustness and reliability of these pooled results (Figure 14B). Furthermore, no publication bias was detected as evidenced by Begg’s test (*p* = 0.787) and Egger’s test (*p* = 0.364) (Figures 14C–E).

#### 3.4.2 Adverse reactions

**FIGURE 15:**
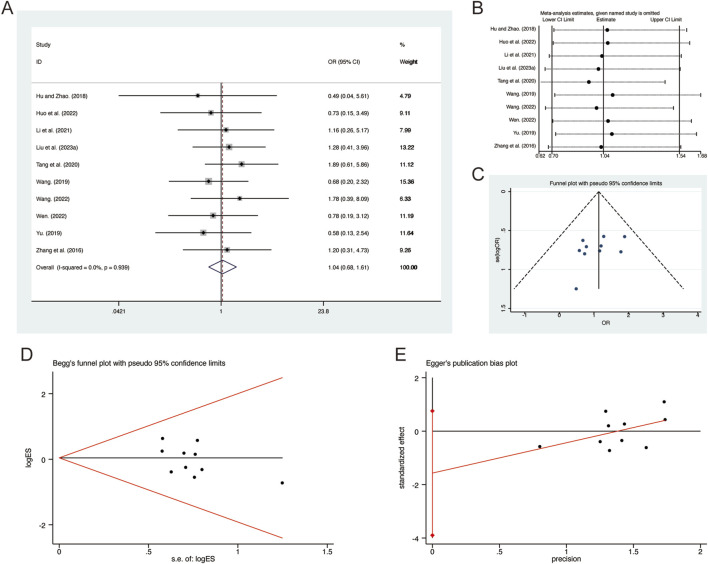
Effect of QHD on adverse reactions in patients with AECOPD. **(A)** Forest plot for adverse reactions. **(B)** Sensitivity analysis for adverse reactions. **(C)** Funnel plot for adverse reactions. **(D)** Begg’s test for adverse reactions. **(E)** Egger’s test for adverse reactions.

A total of 10 studies (Hu and Zhao, 2018; Huo et al., 2022; Li et al., 2021; Liu L. et al., 2023; Tang et al., 2020; Wang, 2019; Wang, 2022; Wen, 2022; Yu, 2019; Zhang et al., 2016) reported on adverse reactions, demonstrating no significant heterogeneity among the studies (*I* ^*2*^ = 0.0%, *p* = 0.939). Consequently, a fixed-effects model was employed to pool the effect sizes. The analysis revealed that, compared to CT, QHD did not increase adverse reactions (RR = 1.04, 95% CI: 0.68 to 1.61, *p* = 0.000, Figure 15A). Sensitivity analysis supported the robustness and reliability of these pooled results (Figure 15B). Furthermore, no publication bias was detected as evidenced by Begg’s test (*p* = 0.283) and Egger’s test (*p* = 0.158) (Figures 15C–E). Common adverse reactions include palpitations, dizziness, headaches, arrhythmias, rashes, liver function abnormalities, nausea, vomiting, diarrhea, abdominal pain, and dry mouth. These adverse reactions were alleviated with symptomatic treatment and did not lead to discontinuation of the study medication. Detailed information on adverse reactions can be found in Supplementary Material S5.

## 4 Discussion

### 4.1 Summary of findings

This meta-analysis is the first to review the effects of QHD on pulmonary function and inflammatory mediators in patients with AECOPD. A total of 40 RCTs involving 3,475 patients were included. The meta-analysis results revealed the following: (1) QHD significantly improved pulmonary function in AECOPD patients, as evidenced by increases in FEV1, FVC, FEV1/FVC, and PaO_2_, and a decrease in PaCO_2_. (2) QHD markedly reduced the inflammatory response in AECOPD patients, indicated by decreased levels of inflammatory mediators such as TNF-α, IL-1β, IL-6, IL-8, CRP, and PCT. (3) In addition to enhancing pulmonary function and reducing inflammatory response, QHD also demonstrated higher clinical efficacy and good safety. Based on these compelling results, our meta-analysis suggests that QHD effectively improves pulmonary function, reduces inflammatory mediators, and enhances overall clinical efficacy in AECOPD patients.

Furthermore, sensitivity analysis confirmed the reliability and robustness of the meta-analysis results by demonstrating that the removal of individual studies did not significantly alter the overall effect sizes. This strengthens confidence in the stability of the findings. In addition to sensitivity analysis, we performed subgroup analyses based on treatment duration. The rationale for choosing treatment duration as a subgroup distinction is based on the hypothesis that longer treatment durations might yield more pronounced effects. Subgroup analysis results indicated that for patients treated with QHD for less than 10 days, improvements in FEV1, FVC, PaCO2, TNF-α, and CRP were more significant compared to those with treatment durations greater than 10 days. Conversely, improvements in FEV1/FVC, PaO2, and overall clinical efficacy were more pronounced in patients with treatment durations exceeding 10 days. However, these differences were not particularly substantial. By examining different subgroups, we were able to explore potential sources of heterogeneity and offer a more nuanced interpretation of the pooled results. To assess potential publication bias, we conducted Begg’s and Egger’s tests, which showed no significant publication bias. This lack of bias further reinforces the reliability and robustness of our findings, as the consistent results across sensitivity and subgroup analyses, combined with the absence of publication bias, provide strong evidence supporting the clinical efficacy and safety of QHD in AECOPD treatment.

### 4.2 Comparison with previous studies

Although Xing et al. (2024) previous meta-analysis explored the clinical efficacy of QHD combined with conventional Western medicine in AECOPD patients, our study specifically focuses on its effects on pulmonary function and inflammatory mediators. Xing et al. primarily examined the short-term clinical outcomes of QHD, such as symptom relief and exacerbation reduction, when used in combination with Western treatments. Their results indicated that QHD, when combined with conventional therapies, significantly reduced the frequency of AECOPD exacerbations and improved clinical efficacy. However, their study did not explore the specific mechanisms underlying these effects, particularly with regard to pulmonary function and inflammatory mediators. In contrast, our study provides a more in-depth analysis of these mechanistic pathways, highlighting significant improvements in FEV1, FVC, FEV1/FVC, and reductions in inflammatory mediators such as TNF-α, IL-1β, and IL-6.

Compared to conventional Western treatments for AECOPD, such as bronchodilators, corticosteroids, and antibiotics, QHD offers a multi-targeted approach by not only improving pulmonary function but also significantly reducing key inflammatory mediators. This dual mechanism of action contrasts with Western treatments, which primarily focus on symptom relief and short-term inflammation control but may not address the underlying pathological changes as comprehensively as QHD. Furthermore, while Western treatments are often associated with adverse effects such as immunosuppression and gastrointestinal discomfort (Brunton and Hogarth, 2023), QHD has demonstrated a favorable safety profile with fewer reported side effects, as evidenced in our meta-analysis and previous studies (Xing et al., 2024). In addition to Western treatments, other herbal interventions have also been explored in AECOPD management. For example, the combination of *Dingchuan* decoction and *Qingqi Huatan* pill has been shown to improve lung function and reduce exacerbation frequency, but studies report that QHD may offer better outcomes for long-term pulmonary function improvement and systemic inflammation reduction (Wu, 2023). Moreover, QHD’s composition, which targets both heat and phlegm in TCM theory, aligns well with the multi-faceted pathology of AECOPD, offering a more holistic approach than some single-component herbal formulas.

In terms of safety, while QHD demonstrated a favorable safety profile in the studies included in our analysis, direct comparisons with Western treatments reveal some advantages. Corticosteroids and bronchodilators, though effective, are often associated with long-term adverse effects such as osteoporosis, hypertension, and increased risk of infections (Bourbeau et al., 2024). In contrast, QHD was associated with fewer and less severe adverse reactions, supporting its potential as a safer alternative or complementary therapy for AECOPD patients. However, the data on adverse events remain limited, and future studies should provide more detailed reports on the nature and severity of adverse reactions to further establish QHD’s safety profile compared to conventional treatments.

### 4.3 Strengths and limitations

The inflammatory response is the main pathological basis of AECOPD (D’Anna et al., 2021). Cigarettes, dust, and toxic gases can induce inflammatory responses, leading to the release of inflammatory mediators such as TNF-α, IL-6, and IL-1β (Chung et al., 2024). These mediators cause airway damage, airflow limitation, and airway remodeling, resulting in decreased pulmonary function and disease progression. Effectively suppressing the inflammatory response is crucial for controlling frequent AECOPD exacerbations and disease progression (Yousuf et al., 2021). This meta-analysis of RCTs is the first to specifically explore the effects of QHD on pulmonary function and inflammatory mediators in AECOPD patients. To enhance the reliability of our findings, we performed subgroup analyses based on different treatment durations to eliminate potential confounding factors related to treatment time. Additionally, we comprehensively assessed the impact of QHD on pulmonary function and inflammatory mediators, highlighting its significance in AECOPD progression, an aspect that previous studies have not fully explored.

This study is not without limitations. Firstly, although we thoroughly searched multiple Chinese and English databases, all the included studies were from China, raising questions about the applicability of the results to other populations. Secondly, the overall quality of the included studies was relatively low. The risk of bias analysis highlighted issues such as unclear randomization methods and insufficient reporting on allocation concealment and blinding, potentially introducing performance and detection biases that compromise internal validity. Although sensitivity and subgroup analyses were conducted, the methodological weaknesses in some studies may have impacted the findings. To reduce bias and improve reliability, future research should ensure standardized and transparent reporting on randomization, allocation, and blinding. Thirdly, the overall sample size of the included studies was small, and none described allocation concealment and blinding. There is an urgent need for large-scale, multicenter, double-blind RCTs to ensure the reliability of the research. Fourthly, the incomplete reporting of both basic study information and safety data, including adverse reactions, drug interactions, and general safety profiles, made it difficult to fully assess QHD’s safety. Strict adherence to clinical research standards and the use of standardized reporting to describe experiments are essential. Fifthly, the number of studies investigating TNF-α, IL-1β, IL-6, IL-8, CRP, and PCT was limited, providing insufficient supporting evidence. Future research should strictly follow clinical research standards and conduct more studies on the effects of QHD on pulmonary function and inflammatory mediators in AECOPD patients to validate our findings. Finally, although our meta-analysis highlighted QHD’s favorable safety profile, the reporting of adverse reactions was somewhat limited. Only a few studies provided detailed descriptions of the nature and severity of adverse reactions. Therefore, future studies should not only focus on the efficacy of QHD but also provide more comprehensive data on its safety, including the nature and long-term implications of any adverse reactions.

### 4.4 Implication

Firstly, future studies should strictly follow clinical research standards with comprehensive, standardized reporting to reduce bias and ensure reliable findings. Additionally, large-scale, multicenter trials across diverse populations are essential to further substantiate and generalize the efficacy of QHD in treating AECOPD. Secondly, alongside efficacy, it is crucial to prioritize detailed safety reporting, including adverse reactions, drug interactions, and long-term safety implications, to provide a comprehensive understanding of QHD’s safety profile. Thirdly, beyond primary outcome measures, future studies should also report the frequency of AECOPD exacerbations, readmission rates, quality of life, and follow-up duration to identify sources of heterogeneity and better clarify patient prognosis. Finally, given the importance of pulmonary function and inflammatory mediators in AECOPD pathogenesis, high-quality research should focus specifically on these aspects.

## 5 Conclusion

In conclusion, this meta-analysis demonstrated that QHD could effectively improve pulmonary function and reduce inflammatory mediators in AECOPD patients, with a good safety profile. However, the included studies exhibited low evidence levels and high heterogeneity, particularly in the assessment of inflammatory mediators. To strengthen these conclusions, future studies should focus on conducting high-quality RCTs.

## Data Availability

The original contributions presented in the study are included in the article/Supplementary Material, further inquiries can be directed to the corresponding authors.
