# Supplementary material for: Effects of Qingjin Huatan decoction on pulmonary function and inflammatory mediators in acute exacerbations of chronic obstructive pulmonary disease: a systematic review and meta-analysis
Source: Front Pharmacol. 2024 Oct 18;15:1466677. doi: 10.3389/fphar.2024.1466677 (PMC11528017; doi:10.3389/fphar.2024.1466677)
Supplement: Supplementary file 2 [file Table1.docx]

Supplementary Material

**Effects of *Qingjin Huatan* Decoction on Pulmonary Function and Inflammatory Mediators in Acute Exacerbations of Chronic Obstructive Pulmonary Disease: A Systematic Review and Meta-analysis**

*Xuqin Du^1^, Yong Chen^2^, Ruodai Zhang^3^, Lipeng Shi^2*^ and Yi Ren^2*^*

*^1^Chongqing University of Chinese Medicine, Chongqing, China*

*^2^Chongqing Traditional Chinese Medicine Hospital, Chongqing, China*

*^3^Chongqing Medical University, Chongqing, China*

*^*^Corresponding authors at: Chongqing Traditional Chinese Medicine Hospital, 6 Panxi Qizhi Road, Jiangbei District, Chongqing, 400021, China.*

*E-mail addresses: lipeng_shi@outlook.com (L. shi). cqszyyzyjdk@163.com (Y. Ren).*

Supplementary Material

[Supplementary Material S1. PRISMA2020·checkliste. 3](#_Toc178931767)

[Supplementary Material S2. Composition and taxonomic authentication of QHD 6](#_Toc178931768)

[Supplementary Material S3. The search strategy. 28](#_Toc178931769)

[Supplementary Material S4. Quality assessment of included studies. 30](#_Toc178931770)

[Supplementary Material S5. The incidence rate of adverse reactions. 33](#_Toc178931771)

# Supplementary Material S1. PRISMA2020·checkliste.

| **Section and Topic** | **Item #** | **Checklist item** | **Location where item is reported** |
| --- | --- | --- | --- |
| **TITLE** | | |  |
| Title | 1 | Identify the report as a systematic review. |  |
| **ABSTRACT** | | |  |
| Abstract | 2 | See the PRISMA 2020 for Abstracts checklist. |  |
| **INTRODUCTION** | | |  |
| Rationale | 3 | Describe the rationale for the review in the context of existing knowledge. |  |
| Objectives | 4 | Provide an explicit statement of the objective(s) or question(s) the review addresses. |  |
| **METHODS** | | |  |
| Eligibility criteria | 5 | Specify the inclusion and exclusion criteria for the review and how studies were grouped for the syntheses. |  |
| Information sources | 6 | Specify all databases, registers, websites, organisations, reference lists and other sources searched or consulted to identify studies. Specify the date when each source was last searched or consulted. |  |
| Search strategy | 7 | Present the full search strategies for all databases, registers and websites, including any filters and limits used. |  |
| Selection process | 8 | Specify the methods used to decide whether a study met the inclusion criteria of the review, including how many reviewers screened each record and each report retrieved, whether they worked independently, and if applicable, details of automation tools used in the process. |  |
| Data collection process | 9 | Specify the methods used to collect data from reports, including how many reviewers collected data from each report, whether they worked independently, any processes for obtaining or confirming data from study investigators, and if applicable, details of automation tools used in the process. |  |
| Data items | 10a | List and define all outcomes for which data were sought. Specify whether all results that were compatible with each outcome domain in each study were sought (e.g. for all measures, time points, analyses), and if not, the methods used to decide which results to collect. |  |
|  | 10b | List and define all other variables for which data were sought (e.g. participant and intervention characteristics, funding sources). Describe any assumptions made about any missing or unclear information. |  |
| Study risk of bias assessment | 11 | Specify the methods used to assess risk of bias in the included studies, including details of the tool(s) used, how many reviewers assessed each study and whether they worked independently, and if applicable, details of automation tools used in the process. |  |
| Effect measures | 12 | Specify for each outcome the effect measure(s) (e.g. risk ratio, mean difference) used in the synthesis or presentation of results. |  |
| Synthesis methods | 13a | Describe the processes used to decide which studies were eligible for each synthesis (e.g. tabulating the study intervention characteristics and comparing against the planned groups for each synthesis (item #5)). |  |
|  | 13b | Describe any methods required to prepare the data for presentation or synthesis, such as handling of missing summary statistics, or data conversions. |  |
|  | 13c | Describe any methods used to tabulate or visually display results of individual studies and syntheses. |  |
|  | 13d | Describe any methods used to synthesize results and provide a rationale for the choice(s). If meta-analysis was performed, describe the model(s), method(s) to identify the presence and extent of statistical heterogeneity, and software package(s) used. |  |
|  | 13e | Describe any methods used to explore possible causes of heterogeneity among study results (e.g. subgroup analysis, meta-regression). |  |
|  | 13f | Describe any sensitivity analyses conducted to assess robustness of the synthesized results. |  |
| Reporting bias assessment | 14 | Describe any methods used to assess risk of bias due to missing results in a synthesis (arising from reporting biases). |  |
| Certainty assessment | 15 | Describe any methods used to assess certainty (or confidence) in the body of evidence for an outcome. |  |
| **RESULTS** | | |  |
| Study selection | 16a | Describe the results of the search and selection process, from the number of records identified in the search to the number of studies included in the review, ideally using a flow diagram. |  |
|  | 16b | Cite studies that might appear to meet the inclusion criteria, but which were excluded, and explain why they were excluded. |  |
| Study characteristics | 17 | Cite each included study and present its characteristics. |  |
| Risk of bias in studies | 18 | Present assessments of risk of bias for each included study. |  |
| Results of individual studies | 19 | For all outcomes, present, for each study: (a) summary statistics for each group (where appropriate) and (b) an effect estimate and its precision (e.g. confidence/credible interval), ideally using structured tables or plots. |  |
| Results of syntheses | 20a | For each synthesis, briefly summarise the characteristics and risk of bias among contributing studies. |  |
|  | 20b | Present results of all statistical syntheses conducted. If meta-analysis was done, present for each the summary estimate and its precision (e.g. confidence/credible interval) and measures of statistical heterogeneity. If comparing groups, describe the direction of the effect. |  |
|  | 20c | Present results of all investigations of possible causes of heterogeneity among study results. |  |
|  | 20d | Present results of all sensitivity analyses conducted to assess the robustness of the synthesized results. |  |
| Reporting biases | 21 | Present assessments of risk of bias due to missing results (arising from reporting biases) for each synthesis assessed. |  |
| Certainty of evidence | 22 | Present assessments of certainty (or confidence) in the body of evidence for each outcome assessed. |  |
| **DISCUSSION** | | |  |
| Discussion | 23a | Provide a general interpretation of the results in the context of other evidence. |  |
|  | 23b | Discuss any limitations of the evidence included in the review. |  |
|  | 23c | Discuss any limitations of the review processes used. |  |
|  | 23d | Discuss implications of the results for practice, policy, and future research. |  |
| **OTHER INFORMATION** | | |  |
| Registration and protocol | 24a | Provide registration information for the review, including register name and registration number, or state that the review was not registered. |  |
|  | 24b | Indicate where the review protocol can be accessed, or state that a protocol was not prepared. |  |
|  | 24c | Describe and explain any amendments to information provided at registration or in the protocol. |  |
| Support | 25 | Describe sources of financial or non-financial support for the review, and the role of the funders or sponsors in the review. |  |
| Competing interests | 26 | Declare any competing interests of review authors. |  |
| Availability of data, code and other materials | 27 | Report which of the following are publicly available and where they can be found: template data collection forms; data extracted from included studies; data used for all analyses; analytic code; any other materials used in the review. |  |

Page MJ, McKenzie JE, Bossuyt PM, et al. The PRISMA 2020 statement: an updated guideline for reporting systematic reviews[J]. BMJ. 2021, 372: n71.

# Supplementary Material S2. Composition and taxonomic authentication of QHD

| Study ID | Chinese name | Species, concentration | Quality control reported? (Y/N) | Chemical analysis reported? (Y/N) |
| --- | --- | --- | --- | --- |
| Chen et al. (2014) | huánɡ qín, 10g | *Scutellaria baicalensis* Georgi. [Lamiaceae; Scutellariae Radix], 10g | N | N |
|  | zhī zǐ, 10g | *Gardenia jasminoides* J.Ellis. [Rubiaceae; Gardeniae Frucyus], 10g | N | N |
|  | jié ɡěnɡ, 10g | *Platycodon grandiflorus* (Jacq.) A.DC. [Campanulaceae; Platycodonis Radix], 10g | N | N |
|  | mài dōnɡ, 10g | *Ophiopogon japonicus* (Thunb.) Ker Gawl. [Asparagaceae; Ophiopogonis Radix], 10g | N | N |
|  | sānɡ bái pí, 15g | *Morus* L. [Moraceae; MoriI Cortex], 15g | N | N |
|  | zhè bèi mǔ, 10g | *Fritillaria thunbergii* Miq. [Liliaceae; Fritillariae Thunbergii Bulbus], 10g | N | N |
|  | zhī mǔ, 10g | *Anemarrhena asphodeloides* Bunge. [Asparagaceae; Anemarrhenae Rhizoma], 10g | N | N |
|  | ɡuā lóu, 15g | *Trichosanthes kirilowii* Maxim. [Cucurbitaceae; Trichosanthis Fructus], 15g | N | N |
|  | tǔ fú línɡ, 15g | *Smilax glabra* Roxb. [Smilacaceae; Smilacis glabrae rhizoma], 15g | N | N |
|  | jú hónɡ, 5g | *Citrus reticulata* Blanco. [Rutaceae; Citri Exocaprium Rubrum], 5g | N | N |
|  | ɡān cǎo, 6g | *Glycyrrhiza uralensis* Fisch. ex DC. [Fabaceae; Glycyrrhizae Radix et Rhizoma], 6g | N | N |
|  | zǐ sū zǐ, 10g | *Perilla frutescens* (L.) Britton. [Lamiaceae; Perillae Fructus], 10g | N | N |
|  | táo rén, 10g | *Prunus persica* (L.) Batsch. [Rosaceae; Persicae Semen], 10g | N | N |
| Chen and Huang. (2015) | huánɡ qín, 10g | *Scutellaria baicalensis* Georgi. [Lamiaceae; Scutellariae Radix], 10g | N | N |
|  | zhī zǐ, 10g | *Gardenia jasminoides* J.Ellis. [Rubiaceae; Gardeniae Frucyus], 10g | N | N |
|  | jié ɡěnɡ, 10g | *Platycodon grandiflorus* (Jacq.) A.DC. [Campanulaceae; Platycodonis Radix], 10g | N | N |
|  | mài dōnɡ, 10g | *Ophiopogon japonicus* (Thunb.) Ker Gawl. [Asparagaceae; Ophiopogonis Radix], 10g | N | N |
|  | sānɡ bái pí, 9g | *Morus* L. [Moraceae; MoriI Cortex], 9g | N | N |
|  | zhè bèi mǔ, 5g | *Fritillaria thunbergii* Miq. [Liliaceae; Fritillariae Thunbergii Bulbus], 5g | N | N |
|  | zhī mǔ, 10g | *Anemarrhena asphodeloides Bunge*. [Asparagaceae; Anemarrhenae Rhizoma], 10g | N | N |
|  | ɡuā lóu, 15g | *Trichosanthes kirilowii* Maxim. [Cucurbitaceae; Trichosanthis Fructus], 15g | N | N |
|  | tǔ fú línɡ, 15g | *Smilax glabra* Roxb. [Smilacaceae; Smilacis glabrae rhizoma], 15g | N | N |
|  | ɡān cǎo, 3g | *Glycyrrhiza uralensis* Fisch. ex DC. [Fabaceae; Glycyrrhizae Radix et Rhizoma], 3g | N | N |
|  | má huánɡ, 5g | *Ephedra sinica* Stapf. [Ephedraceae; Ephedrae Herba], 5g | N | N |
|  | kǔ xìnɡ rén, 10g | *Prunus armeniaca* var. *Armeniaca*. [Rosaceae; Armeniacae Semen Amarum], 10g | N | N |
| Geng et al. (2023) | huánɡ qín, 9g | *Scutellaria baicalensis* Georgi. [Lamiaceae; Scutellariae Radix], 9g | N | N |
|  | zhī zǐ, 9g | *Gardenia jasminoides* J.Ellis. [Rubiaceae; Gardeniae Frucyus], 9g | N | N |
|  | jié ɡěnɡ, 12g | *Platycodon grandiflorus* (Jacq.) A.DC. [Campanulaceae; Platycodonis Radix], 12g | N | N |
|  | mài dōnɡ, 12g | *Ophiopogon japonicus* (Thunb.) Ker Gawl. [Asparagaceae; Ophiopogonis Radix], 12g | N | N |
|  | sānɡ bái pí, 15g | *Morus* L. [Moraceae; MoriI Cortex], 15g | N | N |
|  | zhè bèi mǔ, 10g | *Fritillaria thunbergii* Miq. [Liliaceae; Fritillariae Thunbergii Bulbus], 10g | N | N |
|  | zhī mǔ, 20g | *Anemarrhena asphodeloides* Bunge. [Asparagaceae; Anemarrhenae Rhizoma], 20g | N | N |
|  | ɡuā lóu, 15g | *Trichosanthes kirilowii* Maxim. [Cucurbitaceae; Trichosanthis Fructus], 15g | N | N |
|  | tǔ fú línɡ, 10g | *Smilax glabra* Roxb. [Smilacaceae; Smilacis glabrae rhizoma], 10g | N | N |
|  | jú hónɡ, 12g | *Citrus reticulata* Blanco. [Rutaceae; Citri Exocaprium Rubrum], 12g | N | N |
|  | ɡān cǎo, 6g | *Glycyrrhiza uralensis* Fisch. ex DC. [Fabaceae; Glycyrrhizae Radix et Rhizoma], 6g | N | N |
| Guo. (2018) | huánɡ qín, 10g | *Scutellaria baicalensis* Georgi. [Lamiaceae; Scutellariae Radix], 10g | N | N |
|  | zhī zǐ, 10g | *Gardenia jasminoides* J.Ellis. [Rubiaceae; Gardeniae Frucyus], 10g | N | N |
|  | jié ɡěnɡ, 10g | *Platycodon grandiflorus* (Jacq.) A.DC. [Campanulaceae; Platycodonis Radix], 10g | N | N |
|  | mài dōnɡ, 10g | *Ophiopogon japonicus* (Thunb.) Ker Gawl. [Asparagaceae; Ophiopogonis Radix], 10g | N | N |
|  | sānɡ bái pí, 15g | *Morus* L. [Moraceae; MoriI Cortex], 15g | N | N |
|  | zhè bèi mǔ, 10g | *Fritillaria thunbergii* Miq. [Liliaceae; Fritillariae Thunbergii Bulbus], 10g | N | N |
|  | zhī mǔ, 10g | *Anemarrhena asphodeloides* Bunge. [Asparagaceae; Anemarrhenae Rhizoma], 10g | N | N |
|  | ɡuā lóu, 15g | *Trichosanthes kirilowii* Maxim. [Cucurbitaceae; Trichosanthis Fructus], 15g | N | N |
|  | tǔ fú línɡ, 15g | *Smilax glabra* Roxb. [Smilacaceae; Smilacis glabrae rhizoma], 15g | N | N |
|  | jú hónɡ, 5g | *Citrus reticulata* Blanco. [Rutaceae; Citri Exocaprium Rubrum], 5g | N | N |
|  | ɡān cǎo, 6g | *Glycyrrhiza uralensis* Fisch. ex DC. [Fabaceae; Glycyrrhizae Radix et Rhizoma], 6g | N | N |
|  | zǐ sū zǐ, 10g | *Perilla frutescens* (L.) Britton. [Lamiaceae; Perillae Fructus], 10g | N | N |
|  | táo rén, 10g | *Prunus persica* (L.) Batsch. [Rosaceae; Persicae Semen], 10g | N | N |
| Hu and Zhao. (2018) | huánɡ qín, 12g | *Scutellaria baicalensis* Georgi. [Lamiaceae; Scutellariae Radix], 12g | N | N |
|  | zhī zǐ, 12g | *Gardenia jasminoides* J.Ellis. [Rubiaceae; Gardeniae Frucyus], 12g | N | N |
|  | jié ɡěnɡ, 9g | *Platycodon grandiflorus* (Jacq.) A.DC. [Campanulaceae; Platycodonis Radix], 9g | N | N |
|  | mài dōnɡ, 15g | *Ophiopogon japonicus* (Thunb.) Ker Gawl. [Asparagaceae; Ophiopogonis Radix], 15g | N | N |
|  | sānɡ bái pí, 15g | *Morus* L. [Moraceae; MoriI Cortex], 15g | N | N |
|  | zhè bèi mǔ, 9g | *Fritillaria thunbergii* Miq. [Liliaceae; Fritillariae Thunbergii Bulbus], 9g | N | N |
|  | zhī mǔ, 6g | *Anemarrhena asphodeloides* Bunge. [Asparagaceae; Anemarrhenae Rhizoma], 6g | N | N |
|  | ɡuā lóu, 6g | *Trichosanthes kirilowii* Maxim. [Cucurbitaceae; Trichosanthis Fructus], 6g | N | N |
|  | tǔ fú línɡ, 9g | *Smilax glabra* Roxb. [Smilacaceae; Smilacis glabrae rhizoma], 9g | N | N |
|  | chén pí, 10g | *Citrus reticulata* Blanco. [Rutaceae; Citri Reticulate Pericarpium], 10g | N | N |
|  | ɡān cǎo, 5g | *Glycyrrhiza uralensis* Fisch. ex DC. [Fabaceae; Glycyrrhizae Radix et Rhizoma], 5g | N | N |
| Huang et al. (2022) | huánɡ qín, 12g | *Scutellaria baicalensis* Georgi. [Lamiaceae; Scutellariae Radix], 12g | N | N |
|  | zhī zǐ, 12g | *Gardenia jasminoides* J.Ellis. [Rubiaceae; Gardeniae Frucyus], 12g | N | N |
|  | jié ɡěnɡ, 10g | *Platycodon grandiflorus* (Jacq.) A.DC. [Campanulaceae; Platycodonis Radix], 10g | N | N |
|  | sānɡ bái pí, 20g | *Morus* L. [Moraceae; MoriI Cortex], 20g | N | N |
|  | zhè bèi mǔ, 10g | *Fritillaria thunbergii* Miq. [Liliaceae; Fritillariae Thunbergii Bulbus], 10g | N | N |
|  | ɡuā lóu, 15g | *Trichosanthes kirilowii* Maxim. [Cucurbitaceae; Trichosanthis Fructus]皮, 15g | N | N |
|  | chén pí, 12g | *Citrus reticulata* Blanco. [Rutaceae; Citri Reticulate Pericarpium], 12g | N | N |
|  | ɡān cǎo, 6g | *Glycyrrhiza uralensis* Fisch. ex DC. [Fabaceae; Glycyrrhizae Radix et Rhizoma], 6g | N | N |
|  | má huánɡ, 8g | *Ephedra sinica* Stapf. [Ephedraceae; Ephedrae Herba], 8g | N | N |
|  | zǐ sū zǐ, 12g | *Perilla frutescens* (L.) Britton. [Lamiaceae; Perillae Fructus], 12g | N | N |
| Huo et al. (2022) | huánɡ qín, 10g | *Scutellaria baicalensis* Georgi. [Lamiaceae; Scutellariae Radix], 10g | N | N |
|  | zhī zǐ, 10g | *Gardenia jasminoides* J.Ellis. [Rubiaceae; Gardeniae Frucyus], 10g | N | N |
|  | mài dōnɡ, 10g | *Ophiopogon japonicus* (Thunb.) Ker Gawl. [Asparagaceae; Ophiopogonis Radix], 10g | N | N |
|  | sānɡ bái pí, 10g | *Morus* L. [Moraceae; MoriI Cortex], 10g | N | N |
|  | zhè bèi mǔ, 10g | *Fritillaria thunbergii* Miq. [Liliaceae; Fritillariae Thunbergii Bulbus], 10g | N | N |
|  | zhī mǔ, 10g | *Anemarrhena asphodeloides* Bunge. [Asparagaceae; Anemarrhenae Rhizoma], 10g | N | N |
|  | ɡuā lóu, 10g | *Trichosanthes kirilowii* Maxim. [Cucurbitaceae; Trichosanthis Fructus]皮, 10g | N | N |
|  | tǔ fú línɡ, 10g | *Smilax glabra* Roxb. [Smilacaceae; Smilacis glabrae rhizoma], 10g | N | N |
|  | chén pí, 6g | *Citrus reticulata* Blanco. [Rutaceae; Citri Reticulate Pericarpium], 6g | N | N |
|  | ɡān cǎo, 6g | *Glycyrrhiza uralensis* Fisch. ex DC. [Fabaceae; Glycyrrhizae Radix et Rhizoma], 6g | N | N |
|  | bàn xià, 10g | *Pinellia ternata* (Thunb.) Makino. [Araceae; Pinelliae Rhizoma], 10g | N | N |
|  | kǔ xìnɡ rén, 10g | *Prunus armeniaca* var. *Armeniaca*. [Rosaceae; Armeniacae Semen Amarum], 10g | N | N |
|  | tínɡ lì zǐ, 10g | *Descurainia sophia* (L.) Webb ex Prantl. [Brassicaceae; Descurainiae Semen Lepidii Semen], 10g | N | N |
| Jiang and Liu. (2019) | huánɡ qín, 12g | *Scutellaria baicalensis* Georgi. [Lamiaceae; Scutellariae Radix], 12g | N | N |
|  | zhī zǐ, 12g | *Gardenia jasminoides* J.Ellis. [Rubiaceae; Gardeniae Frucyus], 12g | N | N |
|  | jié ɡěnɡ, 9g | *Platycodon grandiflorus* (Jacq.) A.DC. [Campanulaceae; Platycodonis Radix], 9g | N | N |
|  | mài dōnɡ, 9g | *Ophiopogon japonicus* (Thunb.) Ker Gawl. [Asparagaceae; Ophiopogonis Radix], 9g | N | N |
|  | sānɡ bái pí, 15g | *Morus* L. [Moraceae; MoriI Cortex], 15g | N | N |
|  | zhè bèi mǔ, 9g | *Fritillaria thunbergii* Miq. [Liliaceae; Fritillariae Thunbergii Bulbus], 9g | N | N |
|  | zhī mǔ, 15g | *Anemarrhena asphodeloides* Bunge. [Asparagaceae; Anemarrhenae Rhizoma], 15g | N | N |
|  | ɡuā lóu, 15g | *Trichosanthes kirilowii* Maxim. [Cucurbitaceae; Trichosanthis Fructus], 15g | N | N |
|  | tǔ fú línɡ, 9g | *Smilax glabra* Roxb. [Smilacaceae; Smilacis glabrae rhizoma], 9g | N | N |
|  | jú hónɡ, 9g | *Citrus reticulata* Blanco. [Rutaceae; Citri Exocaprium Rubrum], 9g | N | N |
|  | ɡān cǎo, 3g | *Glycyrrhiza uralensis* Fisch. ex DC. [Fabaceae; Glycyrrhizae Radix et Rhizoma], 3g | N | N |
| Jiang and Chen. (2017) | huánɡ qín, 10g | *Scutellaria baicalensis* Georgi. [Lamiaceae; Scutellariae Radix], 10g | N | N |
|  | zhī zǐ, 10g | *Gardenia jasminoides* J.Ellis. [Rubiaceae; Gardeniae Frucyus], 10g | N | N |
|  | jié ɡěnɡ, 15g | *Platycodon grandiflorus* (Jacq.) A.DC. [Campanulaceae; Platycodonis Radix], 15g | N | N |
|  | mài dōnɡ, 15g | *Ophiopogon japonicus* (Thunb.) Ker Gawl. [Asparagaceae; Ophiopogonis Radix], 15g | N | N |
|  | sānɡ bái pí, 20g | *Morus* L. [Moraceae; MoriI Cortex], 20g | N | N |
|  | zhè bèi mǔ, 10g | *Fritillaria thunbergii* Miq. [Liliaceae; Fritillariae Thunbergii Bulbus], 10g | N | N |
|  | zhī mǔ, 20g | *Anemarrhena asphodeloides* Bunge. [Asparagaceae; Anemarrhenae Rhizoma], 20g | N | N |
|  | ɡuā lóu, 15g | *Trichosanthes kirilowii* Maxim. [Cucurbitaceae; Trichosanthis Fructus], 15g | N | N |
|  | tǔ fú línɡ, 15g | *Smilax glabra* Roxb. [Smilacaceae; Smilacis glabrae rhizoma], 15g | N | N |
|  | chén pí, 15g | *Citrus reticulata* Blanco. [Rutaceae; Citri Reticulate Pericarpium], 15g | N | N |
|  | ɡān cǎo, 10g | *Glycyrrhiza uralensis* Fisch. ex DC. [Fabaceae; Glycyrrhizae Radix et Rhizoma], 10g | N | N |
|  | kǔ xìnɡ rén, 10g | *Prunus armeniaca* var. *Armeniaca*. [Rosaceae; Armeniacae Semen Amarum], 10g | N | N |
|  | bàn xià, 15g | *Pinellia ternata* (Thunb.) Makino. [Araceae; Pinelliae Rhizoma], 15g | N | N |
|  | zǐ sū zǐ, 15g | *Perilla frutescens* (L.) Britton. [Lamiaceae; Perillae Fructus], 15g | N | N |
|  | kuǎn dōnɡ huā, 15g | *Tussilago farfara* L. [Asteraceae; Farfarae Flos], 15g | N | N |
|  | jīn yín huā, 25g | *Lonicera japonica* Thunb. [Caprifoliaceae; Lonicerae Japonicae Flos], 25g | N | N |
|  | lián qiáo, 20g | *Forsythia suspensa* (Thunb.) Vahl. [Oleaceae; Forsythiae Fructus], 20g | N | N |
|  | zǐ yuàn, 20g | *Aster tataricus* L.f. [Asteraceae; Asteris Radix et Rhizoma], 20g | N | N |
| Li et al. (2014) | huánɡ qín, 10g | *Scutellaria baicalensis* Georgi. [Lamiaceae; Scutellariae Radix], 10g | N | N |
|  | zhī zǐ, 10g | *Gardenia jasminoides* J.Ellis. [Rubiaceae; Gardeniae Frucyus], 10g | N | N |
|  | jié ɡěnɡ, 10g | *Platycodon grandiflorus* (Jacq.) A.DC. [Campanulaceae; Platycodonis Radix], 10g | N | N |
|  | mài dōnɡ, 10g | *Ophiopogon japonicus* (Thunb.) Ker Gawl. [Asparagaceae; Ophiopogonis Radix], 10g | N | N |
|  | sānɡ bái pí, 15g | *Morus* L. [Moraceae; MoriI Cortex], 15g | N | N |
|  | zhè bèi mǔ, 10g | *Fritillaria thunbergii* Miq. [Liliaceae; Fritillariae Thunbergii Bulbus], 10g | N | N |
|  | zhī mǔ, 10g | *Anemarrhena asphodeloides* Bunge. [Asparagaceae; Anemarrhenae Rhizoma], 10g | N | N |
|  | ɡuā lóu, 15g | *Trichosanthes kirilowii* Maxim. [Cucurbitaceae; Trichosanthis Fructus], 15g | N | N |
|  | tǔ fú línɡ, 15g | *Smilax glabra* Roxb. [Smilacaceae; Smilacis glabrae rhizoma], 15g | N | N |
|  | jú hónɡ, 5g | *Citrus reticulata* Blanco. [Rutaceae; Citri Exocaprium Rubrum], 5g | N | N |
|  | ɡān cǎo, 6g | *Glycyrrhiza uralensis* Fisch. ex DC. [Fabaceae; Glycyrrhizae Radix et Rhizoma], 6g | N | N |
|  | zǐ sū zǐ, 10g | *Perilla frutescens* (L.) Britton. [Lamiaceae; Perillae Fructus], 10g | N | N |
|  | táo rén, 10g | *Prunus persica* (L.) Batsch. [Rosaceae; Persicae Semen], 10g | N | N |
| Li et al. (2021) | huánɡ qín, 15g | *Scutellaria baicalensis* Georgi. [Lamiaceae; Scutellariae Radix], 15g | N | N |
|  | zhī zǐ, 10g | *Gardenia jasminoides* J.Ellis. [Rubiaceae; Gardeniae Frucyus], 10g | N | N |
|  | sānɡ bái pí, 20g | *Morus* L. [Moraceae; MoriI Cortex], 20g | N | N |
|  | zhè bèi mǔ, 10g | *Fritillaria thunbergii* Miq. [Liliaceae; Fritillariae Thunbergii Bulbus], 10g | N | N |
|  | zhī mǔ, 20g | *Anemarrhena asphodeloides* Bunge. [Asparagaceae; Anemarrhenae Rhizoma], 20g | N | N |
|  | tǔ fú línɡ, 30g | *Smilax glabra* Roxb. [Smilacaceae; Smilacis glabrae rhizoma], 30g | N | N |
|  | ɡān cǎo, 6g | *Glycyrrhiza uralensis* Fisch. ex DC. [Fabaceae; Glycyrrhizae Radix et Rhizoma], 6g | N | N |
|  | tiān nán xīnɡ, 15g | *Arisaema erubescens* (Wall.) Schott. [Araceae; Arisaematis Rhizoma], 15g | N | N |
|  | bàn xià, 15g | *Pinellia ternata* (Thunb.) Makino. [Araceae; Pinelliae Rhizoma], 15g | N | N |
|  | zhǐ shí, 15g | *Citrus* × *aurantium* L. [Rutaceae; Aurantii Fructus Immaturus], 15g | N | N |
|  | kǔ xìnɡ rén, 15g | *Prunus armeniaca* var. *Armeniaca*. [Rosaceae; Armeniacae Semen Amarum], 15g | N | N |
|  | má huánɡ, 10g | *Ephedra sinica* Stapf. [Ephedraceae; Ephedrae Herba], 10g | N | N |
|  | zǐ sū zǐ, 10g | *Perilla frutescens* (L.) Britton. [Lamiaceae; Perillae Fructus], 10g | N | N |
|  | bái ɡuǒ, 15g | *Ginkgo biloba* L. [Ginkgoaceae; Ginkgo Semen], 15g | N | N |
| Liu et al. (2021) | huánɡ qín, 10g | *Scutellaria baicalensis* Georgi. [Lamiaceae; Scutellariae Radix], 10g | N | N |
|  | zhī zǐ, 10g | *Gardenia jasminoides* J.Ellis. [Rubiaceae; Gardeniae Frucyus], 10g | N | N |
|  | jié ɡěnɡ, 6g | *Platycodon grandiflorus* (Jacq.) A.DC. [Campanulaceae; Platycodonis Radix], 6g | N | N |
|  | mài dōnɡ, 10g | *Ophiopogon japonicus* (Thunb.) Ker Gawl. [Asparagaceae; Ophiopogonis Radix], 10g | N | N |
|  | sānɡ bái pí, 10g | *Morus* L. [Moraceae; MoriI Cortex], 10g | N | N |
|  | zhè bèi mǔ, 10g | *Fritillaria thunbergii* Miq. [Liliaceae; Fritillariae Thunbergii Bulbus], 10g | N | N |
|  | zhī mǔ, 10g | *Anemarrhena asphodeloides* Bunge. [Asparagaceae; Anemarrhenae Rhizoma], 10g | N | N |
|  | ɡuā lóu, 10g | *Trichosanthes kirilowii* Maxim. [Cucurbitaceae; Trichosanthis Fructus], 10g | N | N |
|  | tǔ fú línɡ, 10g | *Smilax glabra* Roxb. [Smilacaceae; Smilacis glabrae rhizoma], 10g | N | N |
|  | jú hónɡ, 6g | *Citrus reticulata* Blanco. [Rutaceae; Citri Exocaprium Rubrum], 6g | N | N |
|  | ɡān cǎo, 3g | *Glycyrrhiza uralensis* Fisch. ex DC. [Fabaceae; Glycyrrhizae Radix et Rhizoma], 3g | N | N |
| Liu et al. (2023a) | huánɡ qín, 15g | *Scutellaria baicalensis* Georgi. [Lamiaceae; Scutellariae Radix], 15g | N | N |
|  | zhī zǐ, 15g | *Gardenia jasminoides* J.Ellis. [Rubiaceae; Gardeniae Frucyus], 15g | N | N |
|  | jié ɡěnɡ, 15g | *Platycodon grandiflorus* (Jacq.) A.DC. [Campanulaceae; Platycodonis Radix], 15g | N | N |
|  | mài dōnɡ, 9g | *Ophiopogon japonicus* (Thunb.) Ker Gawl. [Asparagaceae; Ophiopogonis Radix], 9g | N | N |
|  | sānɡ bái pí, 12g | *Morus* L. [Moraceae; MoriI Cortex], 12g | N | N |
|  | zhè bèi mǔ, 9g | *Fritillaria thunbergii* Miq. [Liliaceae; Fritillariae Thunbergii Bulbus], 9g | N | N |
|  | zhī mǔ, 12g | *Anemarrhena asphodeloides* Bunge. [Asparagaceae; Anemarrhenae Rhizoma], 12g | N | N |
|  | ɡuā lóu, 15g | *Trichosanthes kirilowii* Maxim. [Cucurbitaceae; Trichosanthis Fructus], 15g | N | N |
|  | tǔ fú línɡ, 9g | *Smilax glabra* Roxb. [Smilacaceae; Smilacis glabrae rhizoma], 9g | N | N |
|  | jú hónɡ, 15g | *Citrus reticulata* Blanco. [Rutaceae; Citri Exocaprium Rubrum], 15g | N | N |
|  | ɡān cǎo, 9g | *Glycyrrhiza uralensis* Fisch. ex DC. [Fabaceae; Glycyrrhizae Radix et Rhizoma], 9g | N | N |
|  | dān shēn, 15g | *Salvia miltiorrhiza* Bunge. [Lamiaceae; Sal Viae Miltiorrhizae Radix et Rhizoma], 15g | N | N |
| Ni. (2021) | huánɡ qín, 20g | *Scutellaria baicalensis* Georgi. [Lamiaceae; Scutellariae Radix], 20g | N | N |
|  | zhī zǐ, 20g | *Gardenia jasminoides* J.Ellis. [Rubiaceae; Gardeniae Frucyus], 20g | N | N |
|  | mài dōnɡ, 9g | *Ophiopogon japonicus* (Thunb.) Ker Gawl. [Asparagaceae; Ophiopogonis Radix], 9g | N | N |
|  | sānɡ bái pí, 15g | *Morus* L. [Moraceae; MoriI Cortex], 15g | N | N |
|  | zhè bèi mǔ, 9g | *Fritillaria thunbergii* Miq. [Liliaceae; Fritillariae Thunbergii Bulbus], 9g | N | N |
|  | zhī mǔ, 15g | *Anemarrhena asphodeloides* Bunge. [Asparagaceae; Anemarrhenae Rhizoma], 15g | N | N |
|  | ɡuā lóu, 15g | *Trichosanthes kirilowii* Maxim. [Cucurbitaceae; Trichosanthis Fructus], 15g | N | N |
|  | tǔ fú línɡ, 9g | *Smilax glabra* Roxb. [Smilacaceae; Smilacis glabrae rhizoma], 9g | N | N |
|  | jú hónɡ, 9g | *Citrus reticulata* Blanco. [Rutaceae; Citri Exocaprium Rubrum], 9g | N | N |
|  | ɡān cǎo, 3g | *Glycyrrhiza uralensis* Fisch. ex DC. [Fabaceae; Glycyrrhizae Radix et Rhizoma], 3g | N | N |
| Qin. (2022) | huánɡ qín, 10g | *Scutellaria baicalensis* Georgi. [Lamiaceae; Scutellariae Radix], 10g | N | N |
|  | zhī zǐ, 10g | *Gardenia jasminoides* J.Ellis. [Rubiaceae; Gardeniae Frucyus], 10g | N | N |
|  | jié ɡěnɡ, 10g | *Platycodon grandiflorus* (Jacq.) A.DC. [Campanulaceae; Platycodonis Radix], 10g | N | N |
|  | mài dōnɡ, 15g | *Ophiopogon japonicus* (Thunb.) Ker Gawl. [Asparagaceae; Ophiopogonis Radix], 15g | N | N |
|  | sānɡ bái pí, 10g | *Morus* L. [Moraceae; MoriI Cortex], 10g | N | N |
|  | zhè bèi mǔ, 15g | *Fritillaria thunbergii* Miq. [Liliaceae; Fritillariae Thunbergii Bulbus], 15g | N | N |
|  | zhī mǔ, 10g | *Anemarrhena asphodeloides* Bunge. [Asparagaceae; Anemarrhenae Rhizoma], 10g | N | N |
|  | ɡuā lóu, 10g | *Trichosanthes kirilowii* Maxim. [Cucurbitaceae; Trichosanthis Fructus], 10g | N | N |
|  | tǔ fú línɡ, 15g | *Smilax glabra* Roxb. [Smilacaceae; Smilacis glabrae rhizoma], 15g | N | N |
|  | jú hónɡ, 10g | *Citrus reticulata* Blanco. [Rutaceae; Citri Exocaprium Rubrum], 10g | N | N |
|  | ɡān cǎo, 6g | *Glycyrrhiza uralensis* Fisch. ex DC. [Fabaceae; Glycyrrhizae Radix et Rhizoma], 6g | N | N |
|  | kǔ xìnɡ rén, 10g | *Prunus armeniaca* var. *Armeniaca*. [Rosaceae; Armeniacae Semen Amarum], 10g | N | N |
|  | má huánɡ, 10g | *Ephedra sinica* Stapf. [Ephedraceae; Ephedrae Herba], 10g | N | N |
| Sun. (2020) | huánɡ qín, 12g | *Scutellaria baicalensis* Georgi. [Lamiaceae; Scutellariae Radix], 12g | N | N |
|  | zhī zǐ, 12g | *Gardenia jasminoides* J.Ellis. [Rubiaceae; Gardeniae Frucyus], 12g | N | N |
|  | jié ɡěnɡ, 9g | *Platycodon grandiflorus* (Jacq.) A.DC. [Campanulaceae; Platycodonis Radix], 9g | N | N |
|  | mài dōnɡ, 9g | *Ophiopogon japonicus* (Thunb.) Ker Gawl. [Asparagaceae; Ophiopogonis Radix], 9g | N | N |
|  | sānɡ bái pí, 15g | *Morus* L. [Moraceae; MoriI Cortex], 15g | N | N |
|  | zhè bèi mǔ, 9g | *Fritillaria thunbergii* Miq. [Liliaceae; Fritillariae Thunbergii Bulbus], 9g | N | N |
|  | zhī mǔ, 15g | *Anemarrhena asphodeloides* Bunge. [Asparagaceae; Anemarrhenae Rhizoma], 15g | N | N |
|  | ɡuā lóu, 15g | *Trichosanthes kirilowii* Maxim. [Cucurbitaceae; Trichosanthis Fructus], 15g | N | N |
|  | tǔ fú línɡ, 9g | *Smilax glabra* Roxb. [Smilacaceae; Smilacis glabrae rhizoma], 9g | N | N |
|  | jú hónɡ, 9g | *Citrus reticulata* Blanco. [Rutaceae; Citri Exocaprium Rubrum], 9g | N | N |
|  | ɡān cǎo, 3g | *Glycyrrhiza uralensis* Fisch. ex DC. [Fabaceae; Glycyrrhizae Radix et Rhizoma], 3g | N | N |
| Tang et al. (2020) | huánɡ qín, 9g | *Scutellaria baicalensis* Georgi. [Lamiaceae; Scutellariae Radix], 9g | N | N |
|  | zhī zǐ, 12g | *Gardenia jasminoides* J.Ellis. [Rubiaceae; Gardeniae Frucyus], 12g | N | N |
|  | jié ɡěnɡ, 9g | *Platycodon grandiflorus* (Jacq.) A.DC. [Campanulaceae; Platycodonis Radix], 9g | N | N |
|  | mài dōnɡ, 9g | *Ophiopogon japonicus* (Thunb.) Ker Gawl. [Asparagaceae; Ophiopogonis Radix], 9g | N | N |
|  | sānɡ bái pí, 12g | *Morus* L. [Moraceae; MoriI Cortex], 12g | N | N |
|  | zhè bèi mǔ, 9g | *Fritillaria thunbergii* Miq. [Liliaceae; Fritillariae Thunbergii Bulbus], 9g | N | N |
|  | zhī mǔ, 9g | *Anemarrhena asphodeloides* Bunge. [Asparagaceae; Anemarrhenae Rhizoma], 9g | N | N |
|  | ɡuā lóu, 15g | *Trichosanthes kirilowii* Maxim. [Cucurbitaceae; Trichosanthis Fructus], 15g | N | N |
|  | tǔ fú línɡ, 12g | *Smilax glabra* Roxb. [Smilacaceae; Smilacis glabrae rhizoma], 12g | N | N |
|  | ɡān cǎo, 6g | *Glycyrrhiza uralensis* Fisch. ex DC. [Fabaceae; Glycyrrhizae Radix et Rhizoma], 6g | N | N |
|  | táo rén, 9g | *Prunus persica* (L.) Batsch. [Rosaceae; Persicae Semen], 9g | N | N |
| Wang. (2019) | huánɡ qín, 8g | *Scutellaria baicalensis* Georgi. [Lamiaceae; Scutellariae Radix], 8g | N | N |
|  | zhī zǐ, 10g | *Gardenia jasminoides* J.Ellis. [Rubiaceae; Gardeniae Frucyus], 10g | N | N |
|  | jié ɡěnɡ, 9g | *Platycodon grandiflorus* (Jacq.) A.DC. [Campanulaceae; Platycodonis Radix], 9g | N | N |
|  | mài dōnɡ, 10g | *Ophiopogon japonicus* (Thunb.) Ker Gawl. [Asparagaceae; Ophiopogonis Radix], 10g | N | N |
|  | zhè bèi mǔ, 15g | *Fritillaria thunbergii* Miq. [Liliaceae; Fritillariae Thunbergii Bulbus], 15g | N | N |
|  | zhī mǔ, 15g | *Anemarrhena asphodeloides* Bunge. [Asparagaceae; Anemarrhenae Rhizoma], 15g | N | N |
|  | ɡuā lóu, 10g | *Trichosanthes kirilowii* Maxim. [Cucurbitaceae; Trichosanthis Fructus], 10g | N | N |
|  | tǔ fú línɡ, 10g | *Smilax glabra* Roxb. [Smilacaceae; Smilacis glabrae rhizoma], 10g | N | N |
|  | jú hónɡ10g | *Citrus reticulata* Blanco. [Rutaceae; Citri Exocaprium Rubrum]10g | N | N |
|  | ɡān cǎo, 6g | *Glycyrrhiza uralensis* Fisch. ex DC. [Fabaceae; Glycyrrhizae Radix et Rhizoma], 6g | N | N |
|  | kǔ xìnɡ rén, 8g | *Prunus armeniaca* var. *Armeniaca*. [Rosaceae; Armeniacae Semen Amarum], 8g | N | N |
|  | bàn xià, 8g | *Pinellia ternata* (Thunb.) Makino. [Araceae; Pinelliae Rhizoma], 8g | N | N |
|  | zhǐ shí, 6g | *Citrus* × *aurantium* L. [Rutaceae; Aurantii Fructus Immaturus], 6g | N | N |
|  | tiān nán xīnɡ, 5g | *Arisaema erubescens* (Wall.) Schott. [Araceae; Arisaematis Rhizoma], 5g | N | N |
|  | huánɡ lián, 5g | *Coptis chinensis* Franch. [Ranunculaceae; Coptidis Rgizoma], 5g | N | N |
|  | dān shēn, 15g | *Salvia miltiorrhiza* Bunge. [Lamiaceae; Sal Viae Miltiorrhizae Radix et Rhizoma], 15g | N | N |
| Wang. (2022) | huánɡ qín, 10g | *Scutellaria baicalensis* Georgi. [Lamiaceae; Scutellariae Radix], 10g | N | N |
|  | zhī zǐ, 6g | *Gardenia jasminoides* J.Ellis. [Rubiaceae; Gardeniae Frucyus], 6g | N | N |
|  | jié ɡěnɡ, 6g | *Platycodon grandiflorus* (Jacq.) A.DC. [Campanulaceae; Platycodonis Radix], 6g | N | N |
|  | mài dōnɡ, 10g | *Ophiopogon japonicus* (Thunb.) Ker Gawl. [Asparagaceae; Ophiopogonis Radix], 10g | N | N |
|  | sānɡ bái pí, 10g | *Morus* L. [Moraceae; MoriI Cortex], 10g | N | N |
|  | zhè bèi mǔ, 10g | *Fritillaria thunbergii* Miq. [Liliaceae; Fritillariae Thunbergii Bulbus], 10g | N | N |
|  | zhī mǔ, 10g | *Anemarrhena asphodeloides* Bunge. [Asparagaceae; Anemarrhenae Rhizoma], 10g | N | N |
|  | ɡuā lóu, 10g | *Trichosanthes kirilowii* Maxim. [Cucurbitaceae; Trichosanthis Fructus], 10g | N | N |
|  | tǔ fú línɡ, 15g | *Smilax glabra* Roxb. [Smilacaceae; Smilacis glabrae rhizoma], 15g | N | N |
|  | jú hónɡ, 6g | *Citrus reticulata* Blanco. [Rutaceae; Citri Exocaprium Rubrum], 6g | N | N |
|  | ɡān cǎo, 5g | *Glycyrrhiza uralensis* Fisch. ex DC. [Fabaceae; Glycyrrhizae Radix et Rhizoma], 5g | N | N |
| Wei. (2020) | huánɡ qín, 15g | *Scutellaria baicalensis* Georgi. [Lamiaceae; Scutellariae Radix], 15g | N | N |
|  | zhī zǐ, 15g | *Gardenia jasminoides* J.Ellis. [Rubiaceae; Gardeniae Frucyus], 15g | N | N |
|  | jié ɡěnɡ, 10g | *Platycodon grandiflorus* (Jacq.) A.DC. [Campanulaceae; Platycodonis Radix], 10g | N | N |
|  | mài dōnɡ, 10g | *Ophiopogon japonicus* (Thunb.) Ker Gawl. [Asparagaceae; Ophiopogonis Radix], 10g | N | N |
|  | sānɡ bái pí, 15g | *Morus* L. [Moraceae; MoriI Cortex], 15g | N | N |
|  | zhè bèi mǔ, 10g | *Fritillaria thunbergii* Miq. [Liliaceae; Fritillariae Thunbergii Bulbus], 10g | N | N |
|  | zhī mǔ, 15g | *Anemarrhena asphodeloides* Bunge. [Asparagaceae; Anemarrhenae Rhizoma], 15g | N | N |
|  | ɡuā lóu, 15g | *Trichosanthes kirilowii* Maxim. [Cucurbitaceae; Trichosanthis Fructus], 15g | N | N |
|  | tǔ fú línɡ, 10g | *Smilax glabra* Roxb. [Smilacaceae; Smilacis glabrae rhizoma], 10g | N | N |
|  | jú hónɡ, 10g | *Citrus reticulata* Blanco. [Rutaceae; Citri Exocaprium Rubrum], 10g | N | N |
|  | ɡān cǎo, 10g | *Glycyrrhiza uralensis* Fisch. ex DC. [Fabaceae; Glycyrrhizae Radix et Rhizoma], 10g | N | N |
| Wei and Niu. (2017) | huánɡ qín, 10g | *Scutellaria baicalensis* Georgi. [Lamiaceae; Scutellariae Radix], 10g | N | N |
|  | zhī zǐ, 5g | *Gardenia jasminoides* J.Ellis. [Rubiaceae; Gardeniae Frucyus], 5g | N | N |
|  | jié ɡěnɡ, 9g | *Platycodon grandiflorus* (Jacq.) A.DC. [Campanulaceae; Platycodonis Radix], 9g | N | N |
|  | mài dōnɡ, 8g | *Ophiopogon japonicus* (Thunb.) Ker Gawl. [Asparagaceae; Ophiopogonis Radix], 8g | N | N |
|  | sānɡ bái pí, 15g | *Morus* L. [Moraceae; MoriI Cortex], 15g | N | N |
|  | zhè bèi mǔ, 5g | *Fritillaria thunbergii* Miq. [Liliaceae; Fritillariae Thunbergii Bulbus], 5g | N | N |
|  | zhī mǔ, 12g | *Anemarrhena asphodeloides* Bunge. [Asparagaceae; Anemarrhenae Rhizoma], 12g | N | N |
|  | ɡuā lóu, 10g | *Trichosanthes kirilowii* Maxim. [Cucurbitaceae; Trichosanthis Fructus], 10g | N | N |
|  | tǔ fú línɡ, 15g | *Smilax glabra* Roxb. [Smilacaceae; Smilacis glabrae rhizoma], 15g | N | N |
|  | jú hónɡ, 9g | *Citrus reticulata* Blanco. [Rutaceae; Citri Exocaprium Rubrum], 9g | N | N |
|  | ɡān cǎo, 6g | *Glycyrrhiza uralensis* Fisch. ex DC. [Fabaceae; Glycyrrhizae Radix et Rhizoma], 6g | N | N |
| Wen. (2022) | huánɡ qín, 10g | *Scutellaria baicalensis* Georgi. [Lamiaceae; Scutellariae Radix], 10g | N | N |
|  | zhī zǐ, 10g | *Gardenia jasminoides* J.Ellis. [Rubiaceae; Gardeniae Frucyus], 10g | N | N |
|  | jié ɡěnɡ, 10g | *Platycodon grandiflorus* (Jacq.) A.DC. [Campanulaceae; Platycodonis Radix], 10g | N | N |
|  | mài dōnɡ, 10g | *Ophiopogon japonicus* (Thunb.) Ker Gawl. [Asparagaceae; Ophiopogonis Radix], 10g | N | N |
|  | sānɡ bái pí, 15g | *Morus* L. [Moraceae; MoriI Cortex], 15g | N | N |
|  | zhè bèi mǔ, 10g | *Fritillaria thunbergii* Miq. [Liliaceae; Fritillariae Thunbergii Bulbus], 10g | N | N |
|  | zhī mǔ, 10g | *Anemarrhena asphodeloides* Bunge. [Asparagaceae; Anemarrhenae Rhizoma], 10g | N | N |
|  | ɡuā lóu, 15g | *Trichosanthes kirilowii* Maxim. [Cucurbitaceae; Trichosanthis Fructus], 15g | N | N |
|  | tǔ fú línɡ, 15g | *Smilax glabra* Roxb. [Smilacaceae; Smilacis glabrae rhizoma], 15g | N | N |
|  | jú hónɡ, 5g | *Citrus reticulata* Blanco. [Rutaceae; Citri Exocaprium Rubrum], 5g | N | N |
|  | ɡān cǎo, 6g | *Glycyrrhiza uralensis* Fisch. ex DC. [Fabaceae; Glycyrrhizae Radix et Rhizoma], 6g | N | N |
|  | zǐ sū zǐ, 10g | *Perilla frutescens* (L.) Britton. [Lamiaceae; Perillae Fructus], 10g | N | N |
|  | táo rén, 10g | *Prunus persica* (L.) Batsch. [Rosaceae; Persicae Semen], 10g | N | N |
| Wu. (2014) | huánɡ qín, 12g | *Scutellaria baicalensis* Georgi. [Lamiaceae; Scutellariae Radix], 12g | N | N |
|  | zhī zǐ, 12g | *Gardenia jasminoides* J.Ellis. [Rubiaceae; Gardeniae Frucyus], 12g | N | N |
|  | jié ɡěnɡ, 15g | *Platycodon grandiflorus* (Jacq.) A.DC. [Campanulaceae; Platycodonis Radix], 15g | N | N |
|  | mài dōnɡ, 9g | *Ophiopogon japonicus* (Thunb.) Ker Gawl. [Asparagaceae; Ophiopogonis Radix], 9g | N | N |
|  | sānɡ bái pí, 15g | *Morus* L. [Moraceae; MoriI Cortex], 15g | N | N |
|  | zhè bèi mǔ, 9g | *Fritillaria thunbergii* Miq. [Liliaceae; Fritillariae Thunbergii Bulbus], 9g | N | N |
|  | zhī mǔ, 15g | *Anemarrhena asphodeloides* Bunge. [Asparagaceae; Anemarrhenae Rhizoma], 15g | N | N |
|  | ɡuā lóu, 15g | *Trichosanthes kirilowii* Maxim. [Cucurbitaceae; Trichosanthis Fructus], 15g | N | N |
|  | tǔ fú línɡ, 9g | *Smilax glabra* Roxb. [Smilacaceae; Smilacis glabrae rhizoma], 9g | N | N |
|  | jú hónɡ, 9g | *Citrus reticulata* Blanco. [Rutaceae; Citri Exocaprium Rubrum], 9g | N | N |
|  | ɡān cǎo, 6g | *Glycyrrhiza uralensis* Fisch. ex DC. [Fabaceae; Glycyrrhizae Radix et Rhizoma], 6g | N | N |
| Xie. (2016) | huánɡ qín, 12g | *Scutellaria baicalensis* Georgi. [Lamiaceae; Scutellariae Radix], 12g | N | N |
|  | zhī zǐ, 12g | *Gardenia jasminoides* J.Ellis. [Rubiaceae; Gardeniae Frucyus], 12g | N | N |
|  | jié ɡěnɡ, 10g | *Platycodon grandiflorus* (Jacq.) A.DC. [Campanulaceae; Platycodonis Radix], 10g | N | N |
|  | mài dōnɡ, 10g | *Ophiopogon japonicus* (Thunb.) Ker Gawl. [Asparagaceae; Ophiopogonis Radix], 10g | N | N |
|  | sānɡ bái pí, 15g | *Morus* L. [Moraceae; MoriI Cortex], 15g | N | N |
|  | zhè bèi mǔ, 10g | *Fritillaria thunbergii* Miq. [Liliaceae; Fritillariae Thunbergii Bulbus], 10g | N | N |
|  | zhī mǔ, 15g | *Anemarrhena asphodeloides* Bunge. [Asparagaceae; Anemarrhenae Rhizoma], 15g | N | N |
|  | ɡuā lóu, 15g | *Trichosanthes kirilowii* Maxim. [Cucurbitaceae; Trichosanthis Fructus], 15g | N | N |
|  | tǔ fú línɡ, 10g | *Smilax glabra* Roxb. [Smilacaceae; Smilacis glabrae rhizoma], 10g | N | N |
|  | jú hónɡ, 10g | *Citrus reticulata* Blanco. [Rutaceae; Citri Exocaprium Rubrum], 10g | N | N |
|  | ɡān cǎo, 6g | *Glycyrrhiza uralensis* Fisch. ex DC. [Fabaceae; Glycyrrhizae Radix et Rhizoma], 6g | N | N |
| Yang et al. (2023b) | huánɡ qín, 12g | *Scutellaria baicalensis* Georgi. [Lamiaceae; Scutellariae Radix], 12g | N | N |
|  | zhī zǐ, 15g | *Gardenia jasminoides* J.Ellis. [Rubiaceae; Gardeniae Frucyus], 15g | N | N |
|  | jié ɡěnɡ, 12g | *Platycodon grandiflorus* (Jacq.) A.DC. [Campanulaceae; Platycodonis Radix], 12g | N | N |
|  | mài dōnɡ, 12g | *Ophiopogon japonicus* (Thunb.) Ker Gawl. [Asparagaceae; Ophiopogonis Radix], 12g | N | N |
|  | sānɡ bái pí, 15g | *Morus* L. [Moraceae; MoriI Cortex], 15g | N | N |
|  | zhè bèi mǔ, 12g | *Fritillaria thunbergii* Miq. [Liliaceae; Fritillariae Thunbergii Bulbus], 12g | N | N |
|  | zhī mǔ, 15g | *Anemarrhena asphodeloides* Bunge. [Asparagaceae; Anemarrhenae Rhizoma], 15g | N | N |
|  | ɡuā lóu, 30g | *Trichosanthes kirilowii* Maxim. [Cucurbitaceae; Trichosanthis Fructus], 30g | N | N |
|  | tǔ fú línɡ, 15g | *Smilax glabra* Roxb. [Smilacaceae; Smilacis glabrae rhizoma], 15g | N | N |
|  | jú hónɡ, 9g | *Citrus reticulata* Blanco. [Rutaceae; Citri Exocaprium Rubrum], 9g | N | N |
|  | ɡān cǎo, 9g | *Glycyrrhiza uralensis* Fisch. ex DC. [Fabaceae; Glycyrrhizae Radix et Rhizoma], 9g | N | N |
| Yu et al. (2022) | huánɡ qín, 6g | *Scutellaria baicalensis* Georgi. [Lamiaceae; Scutellariae Radix], 6g | N | N |
|  | zhī zǐ, 10g | *Gardenia jasminoides* J.Ellis. [Rubiaceae; Gardeniae Frucyus], 10g | N | N |
|  | mài dōnɡ, 10g | *Ophiopogon japonicus* (Thunb.) Ker Gawl. [Asparagaceae; Ophiopogonis Radix], 10g | N | N |
|  | zhè bèi mǔ, 12g | *Fritillaria thunbergii* Miq. [Liliaceae; Fritillariae Thunbergii Bulbus], 12g | N | N |
|  | zhī mǔ, 12g | *Anemarrhena asphodeloides* Bunge. [Asparagaceae; Anemarrhenae Rhizoma], 12g | N | N |
|  | ɡuā lóu, 15g | *Trichosanthes kirilowii* Maxim. [Cucurbitaceae; Trichosanthis Fructus], 15g | N | N |
|  | ɡān cǎo, 9g | *Glycyrrhiza uralensis* Fisch. ex DC. [Fabaceae; Glycyrrhizae Radix et Rhizoma], 9g | N | N |
|  | bàn xià, 9g | *Pinellia ternata* (Thunb.) Makino. [Araceae; Pinelliae Rhizoma], 9g | N | N |
|  | má huánɡ, 9g | *Ephedra sinica* Stapf. [Ephedraceae; Ephedrae Herba], 9g | N | N |
|  | kǔ xìnɡ rén, 10g | *Prunus armeniaca* var. *Armeniaca*. [Rosaceae; Armeniacae Semen Amarum], 10g | N | N |
|  | zǐ sū zǐ, 9g | *Perilla frutescens* (L.) Britton. [Lamiaceae; Perillae Fructus], 9g | N | N |
|  | kuǎn dōnɡ huā, 9g | *Tussilago farfara* L. [Asteraceae; Farfarae Flos], 9g | N | N |
|  | yú xīnɡ cǎo, 15g | *Houttuynia cordata* Thunb. [Saururaceae; Houttuyniae Herba], 15g | N | N |
| Yu. (2019) | huánɡ qín, 15g | *Scutellaria baicalensis* Georgi. [Lamiaceae; Scutellariae Radix], 15g | N | N |
|  | zhī zǐ, 15g | *Gardenia jasminoides* J.Ellis. [Rubiaceae; Gardeniae Frucyus], 15g | N | N |
|  | jié ɡěnɡ, 15g | *Platycodon grandiflorus* (Jacq.) A.DC. [Campanulaceae; Platycodonis Radix], 15g | N | N |
|  | mài dōnɡ, 10g | *Ophiopogon japonicus* (Thunb.) Ker Gawl. [Asparagaceae; Ophiopogonis Radix], 10g | N | N |
|  | sānɡ bái pí, 12g | *Morus* L. [Moraceae; MoriI Cortex], 12g | N | N |
|  | zhè bèi mǔ, 10g | *Fritillaria thunbergii* Miq. [Liliaceae; Fritillariae Thunbergii Bulbus], 10g | N | N |
|  | zhī mǔ, 12g | *Anemarrhena asphodeloides* Bunge. [Asparagaceae; Anemarrhenae Rhizoma], 12g | N | N |
|  | ɡuā lóu, 15g | *Trichosanthes kirilowii* Maxim. [Cucurbitaceae; Trichosanthis Fructus], 15g | N | N |
|  | tǔ fú línɡ, 10g | *Smilax glabra* Roxb. [Smilacaceae; Smilacis glabrae rhizoma], 10g | N | N |
|  | chén pí, 15g | *Citrus reticulata* Blanco. [Rutaceae; Citri Reticulate Pericarpium], 15g | N | N |
|  | ɡān cǎo, 9g | *Glycyrrhiza uralensis* Fisch. ex DC. [Fabaceae; Glycyrrhizae Radix et Rhizoma], 9g | N | N |
| Yuan. (2018) | huánɡ qín, 10g | *Scutellaria baicalensis* Georgi. [Lamiaceae; Scutellariae Radix], 10g | N | N |
|  | zhī zǐ, 10g | *Gardenia jasminoides* J.Ellis. [Rubiaceae; Gardeniae Frucyus], 10g | N | N |
|  | jié ɡěnɡ, 10g | *Platycodon grandiflorus* (Jacq.) A.DC. [Campanulaceae; Platycodonis Radix], 10g | N | N |
|  | mài dōnɡ, 10g | *Ophiopogon japonicus* (Thunb.) Ker Gawl. [Asparagaceae; Ophiopogonis Radix], 10g | N | N |
|  | sānɡ bái pí, 15g | *Morus* L. [Moraceae; MoriI Cortex], 15g | N | N |
|  | zhè bèi mǔ, 10g | *Fritillaria thunbergii* Miq. [Liliaceae; Fritillariae Thunbergii Bulbus], 10g | N | N |
|  | zhī mǔ, 10g | *Anemarrhena asphodeloides* Bunge. [Asparagaceae; Anemarrhenae Rhizoma], 10g | N | N |
|  | ɡuā lóu, 15g | *Trichosanthes kirilowii* Maxim. [Cucurbitaceae; Trichosanthis Fructus], 15g | N | N |
|  | tǔ fú línɡ, 15g | *Smilax glabra* Roxb. [Smilacaceae; Smilacis glabrae rhizoma], 15g | N | N |
|  | jú hónɡ, 5g | *Citrus reticulata* Blanco. [Rutaceae; Citri Exocaprium Rubrum], 5g | N | N |
|  | ɡān cǎo, 6g | *Glycyrrhiza uralensis* Fisch. ex DC. [Fabaceae; Glycyrrhizae Radix et Rhizoma], 6g | N | N |
|  | zǐ sū zǐ, 10g | *Perilla frutescens* (L.) Britton. [Lamiaceae; Perillae Fructus], 10g | N | N |
| Zhang. (2018a) | huánɡ qín, 10g | *Scutellaria baicalensis* Georgi. [Lamiaceae; Scutellariae Radix], 10g | N | N |
|  | zhī zǐ, 6g | *Gardenia jasminoides* J.Ellis. [Rubiaceae; Gardeniae Frucyus], 6g | N | N |
|  | jié ɡěnɡ, 6g | *Platycodon grandiflorus* (Jacq.) A.DC. [Campanulaceae; Platycodonis Radix], 6g | N | N |
|  | sānɡ bái pí, 3g | *Morus* L. [Moraceae; MoriI Cortex], 3g | N | N |
|  | zhè bèi mǔ, 9g | *Fritillaria thunbergii* Miq. [Liliaceae; Fritillariae Thunbergii Bulbus], 9g | N | N |
|  | zhī mǔ, 6g | *Anemarrhena asphodeloides* Bunge. [Asparagaceae; Anemarrhenae Rhizoma], 6g | N | N |
|  | ɡuā lóu, 3g | *Trichosanthes kirilowii* Maxim. [Cucurbitaceae; Trichosanthis Fructus], 3g | N | N |
|  | tǔ fú línɡ, 4g | *Smilax glabra* Roxb. [Smilacaceae; Smilacis glabrae rhizoma], 4g | N | N |
|  | jú hónɡ, 5g | *Citrus reticulata* Blanco. [Rutaceae; Citri Exocaprium Rubrum], 5g | N | N |
|  | ɡān cǎo, 6g | *Glycyrrhiza uralensis* Fisch. ex DC. [Fabaceae; Glycyrrhizae Radix et Rhizoma], 6g | N | N |
| Zhang. (2018b) | huánɡ qín, 10g | *Scutellaria baicalensis* Georgi. [Lamiaceae; Scutellariae Radix], 10g | N | N |
|  | zhī zǐ, 12g | *Gardenia jasminoides* J.Ellis. [Rubiaceae; Gardeniae Frucyus], 12g | N | N |
|  | jié ɡěnɡ, 9g | *Platycodon grandiflorus* (Jacq.) A.DC. [Campanulaceae; Platycodonis Radix], 9g | N | N |
|  | zhè bèi mǔ, 10g | *Fritillaria thunbergii* Miq. [Liliaceae; Fritillariae Thunbergii Bulbus], 10g | N | N |
|  | zhī mǔ, 10g | *Anemarrhena asphodeloides* Bunge. [Asparagaceae; Anemarrhenae Rhizoma], 10g | N | N |
|  | jú hónɡ, 10g | *Citrus reticulata* Blanco. [Rutaceae; Citri Exocaprium Rubrum], 10g | N | N |
|  | ɡān cǎo, 10g | *Glycyrrhiza uralensis* Fisch. ex DC. [Fabaceae; Glycyrrhizae Radix et Rhizoma], 10g | N | N |
|  | kǔ xìnɡ rén, 10g | *Prunus armeniaca* var. *Armeniaca*. [Rosaceae; Armeniacae Semen Amarum], 10g | N | N |
|  | bǎn lán ɡēn, 20g | *Isatis tinctoria* subsp. Tinctoria. [Brassicaceae; Isatidis Radix], 20g | N | N |
|  | lú ɡēn, 20g | *Phragmites australis* subsp. Australis. [Poaceae; Phragmitis Rhizoma], 20g | N | N |
|  | yú xīnɡ cǎo, 15g | *Houttuynia cordata* Thunb. [Saururaceae; Houttuyniae Herba], 15g | N | N |
|  | bái qián, 10g | *Cynanchum stauntonii* (Decne.) Schltr. ex H.Lév. [Apocynaceae; Cynanchi StauntoniiI Rhizoma Et Radix], 10g | N | N |
| Zhang et al. (2021a) | huánɡ qín, 12g | *Scutellaria baicalensis* Georgi. [Lamiaceae; Scutellariae Radix], 12g | N | N |
|  | zhī zǐ, 12g | *Gardenia jasminoides* J.Ellis. [Rubiaceae; Gardeniae Frucyus], 12g | N | N |
|  | jié ɡěnɡ, 10g | *Platycodon grandiflorus* (Jacq.) A.DC. [Campanulaceae; Platycodonis Radix], 10g | N | N |
|  | sānɡ bái pí, 20g | *Morus* L. [Moraceae; MoriI Cortex], 20g | N | N |
|  | zhè bèi mǔ, 10g | *Fritillaria thunbergii* Miq. [Liliaceae; Fritillariae Thunbergii Bulbus], 10g | N | N |
|  | ɡuā lóu, 15g | *Trichosanthes kirilowii* Maxim. [Cucurbitaceae; Trichosanthis Fructus], 15g | N | N |
|  | jú hónɡ, 12g | *Citrus reticulata* Blanco. [Rutaceae; Citri Exocaprium Rubrum], 12g | N | N |
|  | ɡān cǎo, 6g | *Glycyrrhiza uralensis* Fisch. ex DC. [Fabaceae; Glycyrrhizae Radix et Rhizoma], 6g | N | N |
|  | má huánɡ, 8g | *Ephedra sinica* Stapf. [Ephedraceae; Ephedrae Herba], 8g | N | N |
|  | zǐ sū zǐ, 12g | *Perilla frutescens* (L.) Britton. [Lamiaceae; Perillae Fructus], 12g | N | N |
| Zhang and Li. (2020) | huánɡ qín, 12g | *Scutellaria baicalensis* Georgi. [Lamiaceae; Scutellariae Radix], 12g | N | N |
|  | zhī zǐ, 12g | *Gardenia jasminoides* J.Ellis. [Rubiaceae; Gardeniae Frucyus], 12g | N | N |
|  | jié ɡěnɡ, 10g | *Platycodon grandiflorus* (Jacq.) A.DC. [Campanulaceae; Platycodonis Radix], 10g | N | N |
|  | mài dōnɡ, 10g | *Ophiopogon japonicus* (Thunb.) Ker Gawl. [Asparagaceae; Ophiopogonis Radix], 10g | N | N |
|  | sānɡ bái pí, 15g | *Morus* L. [Moraceae; MoriI Cortex], 15g | N | N |
|  | zhè bèi mǔ, 10g | *Fritillaria thunbergii* Miq. [Liliaceae; Fritillariae Thunbergii Bulbus], 10g | N | N |
|  | zhī mǔ, 10g | *Anemarrhena asphodeloides* Bunge. [Asparagaceae; Anemarrhenae Rhizoma], 10g | N | N |
|  | ɡuā lóu, 15g | *Trichosanthes kirilowii* Maxim. [Cucurbitaceae; Trichosanthis Fructus], 15g | N | N |
|  | tǔ fú línɡ, 10g | *Smilax glabra* Roxb. [Smilacaceae; Smilacis glabrae rhizoma], 10g | N | N |
|  | jú hónɡ, 10g | *Citrus reticulata* Blanco. [Rutaceae; Citri Exocaprium Rubrum], 10g | N | N |
|  | ɡān cǎo, 6g | *Glycyrrhiza uralensis* Fisch. ex DC. [Fabaceae; Glycyrrhizae Radix et Rhizoma], 6g | N | N |
| Zhang and Ge. (2022) | huánɡ qín, 10g | *Scutellaria baicalensis* Georgi. [Lamiaceae; Scutellariae Radix], 10g | N | N |
|  | zhī zǐ, 10g | *Gardenia jasminoides* J.Ellis. [Rubiaceae; Gardeniae Frucyus], 10g | N | N |
|  | jié ɡěnɡ, 10g | *Platycodon grandiflorus* (Jacq.) A.DC. [Campanulaceae; Platycodonis Radix], 10g | N | N |
|  | mài dōnɡ, 15g | *Ophiopogon japonicus* (Thunb.) Ker Gawl. [Asparagaceae; Ophiopogonis Radix], 15g | N | N |
|  | sānɡ bái pí, 10g | *Morus* L. [Moraceae; MoriI Cortex], 10g | N | N |
|  | zhè bèi mǔ, 10g | *Fritillaria thunbergii* Miq. [Liliaceae; Fritillariae Thunbergii Bulbus], 10g | N | N |
|  | ɡuā lóu, 10g | *Trichosanthes kirilowii* Maxim. [Cucurbitaceae; Trichosanthis Fructus], 10g | N | N |
|  | tǔ fú línɡ, 10g | *Smilax glabra* Roxb. [Smilacaceae; Smilacis glabrae rhizoma], 10g | N | N |
|  | jú hónɡ, 15g | *Citrus reticulata* Blanco. [Rutaceae; Citri Exocaprium Rubrum], 15g | N | N |
|  | ɡān cǎo, 6g | *Glycyrrhiza uralensis* Fisch. ex DC. [Fabaceae; Glycyrrhizae Radix et Rhizoma], 6g | N | N |
|  | bàn xià, 10g | *Pinellia ternata* (Thunb.) Makino. [Araceae; Pinelliae Rhizoma], 10g | N | N |
| Zhang et al. (2016) | huánɡ qín, 12g | *Scutellaria baicalensis* Georgi. [Lamiaceae; Scutellariae Radix], 12g | N | N |
|  | zhī zǐ, 12g | *Gardenia jasminoides* J.Ellis. [Rubiaceae; Gardeniae Frucyus], 12g | N | N |
|  | jié ɡěnɡ, 6g | *Platycodon grandiflorus* (Jacq.) A.DC. [Campanulaceae; Platycodonis Radix], 6g | N | N |
|  | mài dōnɡ, 9g | *Ophiopogon japonicus* (Thunb.) Ker Gawl. [Asparagaceae; Ophiopogonis Radix], 9g | N | N |
|  | sānɡ bái pí, 6g | *Morus* L. [Moraceae; MoriI Cortex], 6g | N | N |
|  | zhè bèi mǔ, 9g | *Fritillaria thunbergii* Miq. [Liliaceae; Fritillariae Thunbergii Bulbus], 9g | N | N |
|  | zhī mǔ, 6g | *Anemarrhena asphodeloides* Bunge. [Asparagaceae; Anemarrhenae Rhizoma], 6g | N | N |
|  | ɡuā lóu, 6g | *Trichosanthes kirilowii* Maxim. [Cucurbitaceae; Trichosanthis Fructus], 6g | N | N |
|  | tǔ fú línɡ, 9g | *Smilax glabra* Roxb. [Smilacaceae; Smilacis glabrae rhizoma], 9g | N | N |
|  | jú hónɡ, 9g | *Citrus reticulata* Blanco. [Rutaceae; Citri Exocaprium Rubrum], 9g | N | N |
|  | ɡān cǎo, 5g | *Glycyrrhiza uralensis* Fisch. ex DC. [Fabaceae; Glycyrrhizae Radix et Rhizoma], 5g | N | N |
| Zhang et al. (2015) | huánɡ qín, 12g | *Scutellaria baicalensis* Georgi. [Lamiaceae; Scutellariae Radix], 12g | N | N |
|  | zhī zǐ, 12g | *Gardenia jasminoides* J.Ellis. [Rubiaceae; Gardeniae Frucyus], 12g | N | N |
|  | jié ɡěnɡ, 9g | *Platycodon grandiflorus* (Jacq.) A.DC. [Campanulaceae; Platycodonis Radix], 9g | N | N |
|  | mài dōnɡ, 15g | *Ophiopogon japonicus* (Thunb.) Ker Gawl. [Asparagaceae; Ophiopogonis Radix], 15g | N | N |
|  | sānɡ bái pí, 15g | *Morus* L. [Moraceae; MoriI Cortex], 15g | N | N |
|  | zhè bèi mǔ, 9g | *Fritillaria thunbergii* Miq. [Liliaceae; Fritillariae Thunbergii Bulbus], 9g | N | N |
|  | zhī mǔ, 6g | *Anemarrhena asphodeloides* Bunge. [Asparagaceae; Anemarrhenae Rhizoma], 6g | N | N |
|  | ɡuā lóu, 6g | *Trichosanthes kirilowii* Maxim. [Cucurbitaceae; Trichosanthis Fructus], 6g | N | N |
|  | tǔ fú línɡ, 9g | *Smilax glabra* Roxb. [Smilacaceae; Smilacis glabrae rhizoma], 9g | N | N |
|  | ɡān cǎo, 5g | *Glycyrrhiza uralensis* Fisch. ex DC. [Fabaceae; Glycyrrhizae Radix et Rhizoma], 5g | N | N |
| Zhao. (2016) | huánɡ qín, 15g | *Scutellaria baicalensis* Georgi. [Lamiaceae; Scutellariae Radix], 15g | N | N |
|  | zhī zǐ, 15g | *Gardenia jasminoides* J.Ellis. [Rubiaceae; Gardeniae Frucyus], 15g | N | N |
|  | jié ɡěnɡ, 15g | *Platycodon grandiflorus* (Jacq.) A.DC. [Campanulaceae; Platycodonis Radix], 15g | N | N |
|  | mài dōnɡ, 10g | *Ophiopogon japonicus* (Thunb.) Ker Gawl. [Asparagaceae; Ophiopogonis Radix], 10g | N | N |
|  | sānɡ bái pí, 12g | *Morus* L. [Moraceae; MoriI Cortex], 12g | N | N |
|  | zhè bèi mǔ, 10g | *Fritillaria thunbergii* Miq. [Liliaceae; Fritillariae Thunbergii Bulbus], 10g | N | N |
|  | zhī mǔ, 12g | *Anemarrhena asphodeloides* Bunge. [Asparagaceae; Anemarrhenae Rhizoma], 12g | N | N |
|  | ɡuā lóu, 15g | *Trichosanthes kirilowii* Maxim. [Cucurbitaceae; Trichosanthis Fructus], 15g | N | N |
|  | tǔ fú línɡ, 10g | *Smilax glabra* Roxb. [Smilacaceae; Smilacis glabrae rhizoma], 10g | N | N |
|  | chén pí, 15g | *Citrus reticulata* Blanco. [Rutaceae; Citri Reticulate Pericarpium], 15g | N | N |
|  | ɡān cǎo, 10g | *Glycyrrhiza uralensis* Fisch. ex DC. [Fabaceae; Glycyrrhizae Radix et Rhizoma], 10g | N | N |
| Zhao. (2023) | huánɡ qín, 12g | *Scutellaria baicalensis* Georgi. [Lamiaceae; Scutellariae Radix], 12g | N | N |
|  | zhī zǐ, 12g | *Gardenia jasminoides* J.Ellis. [Rubiaceae; Gardeniae Frucyus], 12g | N | N |
|  | jié ɡěnɡ, 10g | *Platycodon grandiflorus* (Jacq.) A.DC. [Campanulaceae; Platycodonis Radix], 10g | N | N |
|  | sānɡ bái pí, 20g | *Morus* L. [Moraceae; MoriI Cortex], 20g | N | N |
|  | zhè bèi mǔ, 10g | *Fritillaria thunbergii* Miq. [Liliaceae; Fritillariae Thunbergii Bulbus], 10g | N | N |
|  | ɡuā lóu, 15g | *Trichosanthes kirilowii* Maxim. [Cucurbitaceae; Trichosanthis Fructus], 15g | N | N |
|  | chén pí, 12g | *Citrus reticulata* Blanco. [Rutaceae; Citri Reticulate Pericarpium], 12g | N | N |
|  | ɡān cǎo, 6g | *Glycyrrhiza uralensis* Fisch. ex DC. [Fabaceae; Glycyrrhizae Radix et Rhizoma], 6g | N | N |
|  | má huánɡ, 8g | *Ephedra sinica* Stapf. [Ephedraceae; Ephedrae Herba], 8g | N | N |
|  | zǐ sū zǐ, 12g | *Perilla frutescens* (L.) Britton. [Lamiaceae; Perillae Fructus], 12g | N | N |
| Zhou et al. (2014) | huánɡ qín, 6g | *Scutellaria baicalensis* Georgi. [Lamiaceae; Scutellariae Radix], 6g | N | N |
|  | zhī zǐ, 9g | *Gardenia jasminoides* J.Ellis. [Rubiaceae; Gardeniae Frucyus], 9g | N | N |
|  | jié ɡěnɡ, 6g | *Platycodon grandiflorus* (Jacq.) A.DC. [Campanulaceae; Platycodonis Radix], 6g | N | N |
|  | mài dōnɡ, 10g | *Ophiopogon japonicus* (Thunb.) Ker Gawl. [Asparagaceae; Ophiopogonis Radix], 10g | N | N |
|  | zhè bèi mǔ, 5g | *Fritillaria thunbergii* Miq. [Liliaceae; Fritillariae Thunbergii Bulbus], 5g | N | N |
|  | zhī mǔ, 5g | *Anemarrhena asphodeloides* Bunge. [Asparagaceae; Anemarrhenae Rhizoma], 5g | N | N |
|  | ɡuā lóu, 10g | *Trichosanthes kirilowii* Maxim. [Cucurbitaceae; Trichosanthis Fructus], 10g | N | N |
|  | tǔ fú línɡ, 10g | *Smilax glabra* Roxb. [Smilacaceae; Smilacis glabrae rhizoma], 10g | N | N |
|  | chén pí, 10g | *Citrus reticulata* Blanco. [Rutaceae; Citri Reticulate Pericarpium], 10g | N | N |
|  | ɡān cǎo, 6g | *Glycyrrhiza uralensis* Fisch. ex DC. [Fabaceae; Glycyrrhizae Radix et Rhizoma], 6g | N | N |
|  | tiān nán xīnɡ, 3g | *Arisaema erubescens* (Wall.) Schott. [Araceae; Arisaematis Rhizoma], 3g | N | N |
|  | bàn xià, 6g | *Pinellia ternata* (Thunb.) Makino. [Araceae; Pinelliae Rhizoma], 6g | N | N |
|  | huánɡ lián, 3g | *Coptis chinensis* Franch. [Ranunculaceae; Coptidis Rgizoma], 3g | N | N |
|  | kǔ xìnɡ rén, 6g | *Prunus armeniaca* var. *Armeniaca*. [Rosaceae; Armeniacae Semen Amarum], 6g | N | N |
|  | zhǐ shí, 6g | *Citrus* × *aurantium* L. [Rutaceae; Aurantii Fructus Immaturus], 6g | N | N |
| Zhou et al. (2016) | huánɡ qín, 10g | *Scutellaria baicalensis* Georgi. [Lamiaceae; Scutellariae Radix], 10g | N | N |
|  | zhī zǐ, 10g | *Gardenia jasminoides* J.Ellis. [Rubiaceae; Gardeniae Frucyus], 10g | N | N |
|  | jié ɡěnɡ, 10g | *Platycodon grandiflorus* (Jacq.) A.DC. [Campanulaceae; Platycodonis Radix], 10g | N | N |
|  | mài dōnɡ, 10g | *Ophiopogon japonicus* (Thunb.) Ker Gawl. [Asparagaceae; Ophiopogonis Radix], 10g | N | N |
|  | sānɡ bái pí, 20g | *Morus* L. [Moraceae; MoriI Cortex], 20g | N | N |
|  | zhī mǔ, 10g | *Anemarrhena asphodeloides* Bunge. [Asparagaceae; Anemarrhenae Rhizoma], 10g | N | N |
|  | ɡuā lóu, 20g | *Trichosanthes kirilowii* Maxim. [Cucurbitaceae; Trichosanthis Fructus], 20g | N | N |
|  | chén pí, 10g | *Citrus reticulata* Blanco. [Rutaceae; Citri Reticulate Pericarpium], 10g | N | N |
|  | ɡān cǎo, 6g | *Glycyrrhiza uralensis* Fisch. ex DC. [Fabaceae; Glycyrrhizae Radix et Rhizoma], 6g | N | N |
|  | bàn xià, 6g | *Pinellia ternata* (Thunb.) Makino. [Araceae; Pinelliae Rhizoma], 6g | N | N |
|  | zǐ sū zǐ, 10g | *Perilla frutescens* (L.) Britton. [Lamiaceae; Perillae Fructus], 10g | N | N |
|  | kǔ xìnɡ rén, 10g | *Prunus armeniaca* var. *Armeniaca*. [Rosaceae; Armeniacae Semen Amarum], 10g | N | N |
| Zhu. (2021) | huánɡ qín, 12g | *Scutellaria baicalensis* Georgi. [Lamiaceae; Scutellariae Radix], 12g | N | N |
|  | zhī zǐ, 12g | *Gardenia jasminoides* J.Ellis. [Rubiaceae; Gardeniae Frucyus], 12g | N | N |
|  | jié ɡěnɡ, 9g | *Platycodon grandiflorus* (Jacq.) A.DC. [Campanulaceae; Platycodonis Radix], 9g | N | N |
|  | mài dōnɡ, 9g | *Ophiopogon japonicus* (Thunb.) Ker Gawl. [Asparagaceae; Ophiopogonis Radix], 9g | N | N |
|  | sānɡ bái pí, 15g | *Morus* L. [Moraceae; MoriI Cortex], 15g | N | N |
|  | zhè bèi mǔ, 9g | *Fritillaria thunbergii* Miq. [Liliaceae; Fritillariae Thunbergii Bulbus], 9g | N | N |
|  | zhī mǔ, 15g | *Anemarrhena asphodeloides* Bunge. [Asparagaceae; Anemarrhenae Rhizoma], 15g | N | N |
|  | ɡuā lóu, 15g | *Trichosanthes kirilowii* Maxim. [Cucurbitaceae; Trichosanthis Fructus], 15g | N | N |
|  | tǔ fú línɡ, 9g | *Smilax glabra* Roxb. [Smilacaceae; Smilacis glabrae rhizoma], 9g | N | N |
|  | jú hónɡ, 9g | *Citrus reticulata* Blanco. [Rutaceae; Citri Exocaprium Rubrum], 9g | N | N |
|  | ɡān cǎo, 3g | *Glycyrrhiza uralensis* Fisch. ex DC. [Fabaceae; Glycyrrhizae Radix et Rhizoma], 3g | N | N |
|  |  |  |  |  |

#

# Supplementary Material S3. The search strategy.

**Search run on May 31 2024**

**PubMed (*n*=6)**

#1 ((Qingjin Huatan Decoction [Title/Abstract]) OR (Qingjin Huatan Tang [Title/Abstract]))

#2 ((acute exacerbations of chronic obstructive pulmonary disease [Title/Abstract]) OR (AECOPD [Title/Abstract]) OR (chronic obstructive pulmonary disease [Title/Abstract]) OR (COPD [Title/Abstract]))

#3 #1 AND #2

**Embase (*n*=3)**

#1 'Qingjin Huatan Decoction'/exp

#2 'Qingjin Huatan Tang'/exp

#3 #1 OR #2

#4 'acute exacerbations of chronic obstructive pulmonary disease'/exp

#5 'AECOPD'/exp

#6 'chronic obstructive pulmonary disease'/exp

#7 'COPD'/exp

#8 #4 OR #5 OR #6 OR #7

#9 #3 AND #8

**Cochrane library (*n*=4)**

#1 MeSH descriptor: [Qingjin Huatan Decoction] explode all trees

#2 Qingjin Huatan Tang* or Qingjin Huatan*.ti,ab,kw

#3 #1 or #2

#4 MeSH descriptor: [acute exacerbations of chronic obstructive pulmonary disease] explode all trees

#5 chronic obstructive pulmonary disease* or AECOPD* or COPD*.ti,ab,kw

#6 #4 or #5

#7 #3 and #6

**Web of Science (*n*=3)**

#1 (TS=(Qingjin Huatan Decoction) OR ALL=(Qingjin Huatan Tang))

#2 (TS=(acute exacerbations of chronic obstructive pulmonary disease) OR ALL=(chronic obstructive pulmonary disease) OR ALL=(AECOPD) OR ALL=(COPD))

#3 #1 AND #2

**CNKI (*n*=142)**

#1 篇关摘：清金化痰汤 OR 清金化痰颗粒

#2 篇关摘：慢性阻塞性肺疾病急性加重 OR 慢性阻塞性肺疾病 OR AECOPD OR COPD OR 慢阻肺

#3 #1 AND #2

**Wanfang Data (*n*=143)**

#1 主题：清金化痰汤 OR 清金化痰颗粒

#2 主题：慢性阻塞性肺疾病急性加重 OR 慢性阻塞性肺疾病 OR AECOPD OR COPD OR 慢阻肺

#3 #1 AND #2

**CQVIP (*n*=96)**

#1 篇关摘：清金化痰汤 OR 清金化痰颗粒

#2 篇关摘：慢性阻塞性肺疾病急性加重 OR 慢性阻塞性肺疾病 OR AECOPD OR COPD OR 慢阻肺

#3 #1 AND #2

**CBM (*n*=117)**

#1 常用字段：清金化痰汤 OR 清金化痰颗粒

#2 常用字段：慢性阻塞性肺疾病急性加重 OR 慢性阻塞性肺疾病 OR AECOPD OR COPD OR 慢阻肺

#3 #1 AND #2

**中国临床试验注册中心 (*n*=2)**

# Supplementary Material S4. Quality assessment of included studies.

| Study ID | Random sequence generation | Allocation concealment | Blinding | | Incomplete outcome data | Selective reporting | Other biases | Modified Jadad scores |
| --- | --- | --- | --- | --- | --- | --- | --- | --- |
|  |  |  | Blinding of participants and personnel | Blinding of outcome assessment |  |  |  |  |
| Chen et al. (2014) | Unclear risk | Unclear risk | Unclear risk | Unclear risk | Low risk | Low risk | Unclear risk | 3 |
| Chen and Huang. (2015) | High risk | Unclear risk | Unclear risk | Unclear risk | Low risk | Low risk | Unclear risk | 2 |
| Geng et al. (2023) | Low risk | Unclear risk | Unclear risk | Unclear risk | Low risk | Low risk | Unclear risk | 4 |
| Guo. (2018) | Low risk | Unclear risk | Unclear risk | Unclear risk | Low risk | Low risk | Unclear risk | 4 |
| Hu and Zhao. (2018) | Low risk | Unclear risk | Unclear risk | Unclear risk | Low risk | Low risk | Unclear risk | 4 |
| Huang et al. (2022) | Low risk | Unclear risk | Unclear risk | Unclear risk | Low risk | Low risk | Unclear risk | 4 |
| Huo et al. (2022) | Low risk | Unclear risk | Unclear risk | Unclear risk | Low risk | Low risk | Unclear risk | 4 |
| Jiang and Liu. (2019) | Low risk | Unclear risk | Unclear risk | Unclear risk | Low risk | Low risk | Unclear risk | 4 |
| Jiang and Chen. (2017) | Low risk | Unclear risk | Unclear risk | Unclear risk | Low risk | Low risk | Unclear risk | 4 |
| Li et al. (2014) | Low risk | Unclear risk | Unclear risk | Unclear risk | Low risk | Low risk | Unclear risk | 4 |
| Li et al. (2021) | High risk | Unclear risk | Unclear risk | Unclear risk | Low risk | Low risk | Unclear risk | 2 |
| Liu et al. (2021) | Low risk | Unclear risk | Unclear risk | Unclear risk | Low risk | Low risk | Unclear risk | 4 |
| Liu et al. (2023a) | Low risk | Unclear risk | Unclear risk | Unclear risk | Low risk | Low risk | Unclear risk | 4 |
| Ni. (2021) | Low risk | Unclear risk | Unclear risk | Unclear risk | Low risk | Low risk | Unclear risk | 4 |
| Qin. (2022) | Low risk | Unclear risk | Unclear risk | Unclear risk | Low risk | Low risk | Unclear risk | 4 |
| Sun. (2020) | Low risk | Unclear risk | Unclear risk | Unclear risk | Low risk | Low risk | Unclear risk | 4 |
| Tang et al. (2020) | Low risk | Unclear risk | Unclear risk | Unclear risk | Low risk | Low risk | Unclear risk | 4 |
| Wang. (2019) | Low risk | Unclear risk | Unclear risk | Unclear risk | Low risk | Low risk | Unclear risk | 4 |
| Wang. (2022) | Low risk | Unclear risk | Unclear risk | Unclear risk | Low risk | Low risk | Unclear risk | 4 |
| Wei. (2020) | Low risk | Unclear risk | Unclear risk | Unclear risk | Low risk | Low risk | Unclear risk | 4 |
| Wei and Niu. (2017) | Unclear risk | Unclear risk | Unclear risk | Unclear risk | Low risk | Low risk | Unclear risk | 3 |
| Wen. (2022) | Low risk | Unclear risk | Unclear risk | Unclear risk | Low risk | Low risk | Unclear risk | 4 |
| Wu. (2014) | Low risk | Unclear risk | Unclear risk | Unclear risk | Low risk | Low risk | Unclear risk | 4 |
| Xie. (2016) | Unclear risk | Unclear risk | Unclear risk | Unclear risk | Low risk | Low risk | Unclear risk | 3 |
| Yang et al. (2023b) | Low risk | Unclear risk | Unclear risk | Unclear risk | Low risk | Low risk | Unclear risk | 4 |
| Yu et al. (2022) | Low risk | Unclear risk | Unclear risk | Unclear risk | Low risk | Low risk | Unclear risk | 4 |
| Yu. (2019) | Low risk | Unclear risk | Unclear risk | Unclear risk | Low risk | Low risk | Unclear risk | 4 |
| Yuan. (2018) | Unclear risk | Unclear risk | Unclear risk | Unclear risk | Low risk | Low risk | Unclear risk | 3 |
| Zhang. (2018a) | Unclear risk | Unclear risk | Unclear risk | Unclear risk | Low risk | Low risk | Unclear risk | 3 |
| Zhang. (2018b) | Low risk | Unclear risk | Unclear risk | Unclear risk | Low risk | Low risk | Unclear risk | 4 |
| Zhang et al. (2021a) | Unclear risk | Unclear risk | Unclear risk | Unclear risk | Low risk | Low risk | Unclear risk | 3 |
| Zhang and Li. (2020) | Low risk | Unclear risk | Unclear risk | Unclear risk | Low risk | Low risk | Unclear risk | 4 |
| Zhang and Ge. (2022) | Low risk | Unclear risk | Unclear risk | Unclear risk | Low risk | Low risk | Unclear risk | 4 |
| Zhang et al. (2016) | Low risk | Unclear risk | Unclear risk | Unclear risk | Low risk | Low risk | Unclear risk | 4 |
| Zhang et al. (2015) | Unclear risk | Unclear risk | Unclear risk | Unclear risk | Low risk | Low risk | Unclear risk | 3 |
| Zhao. (2016) | Low risk | Unclear risk | Unclear risk | Unclear risk | Low risk | Low risk | Unclear risk | 4 |
| Zhao. (2023) | Low risk | Unclear risk | Unclear risk | Unclear risk | Low risk | Low risk | Unclear risk | 4 |
| Zhou et al. (2014) | Unclear risk | Unclear risk | Unclear risk | Unclear risk | Low risk | Low risk | Unclear risk | 3 |
| Zhou et al. (2016) | Low risk | Unclear risk | Unclear risk | Unclear risk | Low risk | Low risk | Unclear risk | 4 |
| Zhu. (2021) | Low risk | Unclear risk | Unclear risk | Unclear risk | Low risk | Low risk | Unclear risk | 4 |

# Supplementary Material S5. The incidence rate of adverse reactions.

| Adverse reaction symptoms | First author  (publication year) | The number of adverse reactions | |
| --- | --- | --- | --- |
|  |  | T | C |
| Palpitation | Liu et al. (2023a); Wang. (2019); Wen. (2022); Yu. (2019) | 4 | 8 |
| Dizziness | Tang et al. (2020) | 3 | 2 |
| Headache | Li et al. (2021); Liu et al. (2023) | 5 | 5 |
| Arrhythmia | Wang. (2022); Zhang et al. (2016) | 1 | 3 |
| Rash | Hu and Zhao. (2018); Tang et al. (2020); Wang. (2022); Zhang et al. (2016) | 5 | 1 |
| Abnormal liver function | Hu and Zhao. (2018) | 1 | 0 |
| Nausea | Huo et al. (2022); Liu et al. (2023); Tang et al. (2020); Wang. (2019); Wang. (2022); Wen. (2022); Zhang et al. (2016) | 16 | 14 |
| Vomit | Li et al. (2021) | 1 | 0 |
| Diarrhea | Hu and Zhao. (2018); Wang. (2019); Wen. (2022); Yu. (2019) | 5 | 4 |
| Abdominal pain | Huo et al. (2022) | 1 | 2 |
| Dry mouth | Tang et al. (2020); Wang. (2022); Yu. (2019); Zhang et al. (2016) | 6 | 5 |
| Total reactions | － | 48/526 | 44/501 |
| Incidence rate | － | 9.13% | 8.78% |
